# A highly diverse Pennsylvanian tetrapod ichnoassemblage from the Semily Formation (Krkonoše Piedmont Basin, Czechia)

**DOI:** 10.7717/peerj.20437

**Published:** 2026-01-09

**Authors:** Gabriela Calábková, Jakub Březina, Roland Nádaskay

**Affiliations:** 1 Department of Geological Sciences, Faculty of Science, Masaryk University, Brno, Czech Republic; 2 Department of Geology and Paleontology, Moravian Museum, Brno, Czech Republic; 3 Czech Geological Survey, Prague, Czech Republic

**Keywords:** Tetrapoda, Footprints, Gzhelian, Ploužnice Horizon, Ploužnice Lake

## Abstract

The Krkonoše Piedmont Basin (KPB) is one of the Late Paleozoic continental basins in Bohemia, Czechia, comprising a sedimentary sequence from the Late Pennsylvanian to the early Cisuralian. The Pennsylvanian in the KPB consists of alluvial-fluvial to lacustrine deposits with a relatively rich fossil record, comprising mainly ray-finned fishes, freshwater sharks, and invertebrates. Although no skeletal remains of terrestrial vertebrates have been discovered in the Late Pennsylvanian deposits of the KPB, recent studies of tetrapod footprints provide the first direct evidence of pre-Permian terrestrial tetrapod diversity within this basin. A diverse ichnofossil assemblage is represented by six ichnogenera, *Amphisauropus*, *Batrachichnus*, *Dimetropu*s, *Dromopus*, *Ichniotherium* and *Limnopus*, including five known ichnospecies, *Amphisauropus kablikae*, *Batrachichnus salamandroides*, *Dromopus lacertoides, Ichniotherium cottae*, and *Limnopus heterodactylus*, and two unknown ichnospecies, *Dimetropus* isp. and *Limnopus* isp. This tetrapod ichnoassemblage is among the most diverse in the Pennsylvanian. Moreover, the *Amphisauropus* tracks from the KPB represent the first undisputed occurrence of this ichnotaxon in the Gzhelian. Furthermore, the *Ichniotherium cottae* tracks described here complement the still rare Pennsylvanian occurrences of this ichnospecies in the European part of Pangaea. The ichnofauna studied herein is associated with alluvial-plain to lacustrine nearshore deposits, highlighting the ecological importance of the lacustrine environment and its adjacent areas for the presence of terrestrial vertebrates and the preservation of their footprints.

## Introduction

The Krkonoše Piedmont Basin (KPB) preserves the most abundant Pennsylvanian tetrapod footprint assemblage in Czechia, offering invaluable insights into the early tetrapod diversity of a landlocked basin that formed part of equatorial Pangaea. Tetrapod footprint localities in the KBP have been documented since the 19th century and hold historical significance primarily due to the early Permian locality of Horní Kalná (Prosečné Formation), which served as the type locality for three typical ichnospecies of the Late Paleozoic (see Geinitz, 1861; Geinitz & Deichmüller, 1882). However, finds of Pennsylvanian tetrapod tracks have until recently been described much less frequently in this area. Frič (1912a) and Frič (1912b) was the first to report the occurrences of *Saurichnites calcaratus*, a junior synonym of *Dromopus lacertoides* (Geinitz, 1861) in the railway cut Kyje–Ploužnice (Ploužnice Horizon, Gzhelian) in the KPB. Later, Haubold & Katzung (1975) expanded the ichnotaxonomic list from the same locality with *Anthichnium salamandroides*, a junior synonym of *Batrachichnus salamandroides*, (Geinitz, 1861), *Amphisauropus latus*, a junior synonym of *Amphisauropus kablikae*, (Geinitz & Deichmüller, 1882), *Dromopus lacertoides* (Geinitz, 1861), and *Ichniotherium cottae* (Pohlig, 1885). However, these early studies lacked descriptions or depictions of the listed ichnotaxa. Despite this, the relatively diverse tetrapod ichnoassemblage contrasts sharply with the extremely rare tetrapod body fossils in the Pennsylvanian of the KPB, which are limited to a few bones of branchiosaurid temnospondyls (Frič, 1912a; Frič, 1912b).

This study presents the first comprehensive description of Pennsylvanian tetrapod tracks from the KPB, based on new fossil finds—including the first record of tracks from the lacustrine Ploužnice Horizon of the Semily Fm. (Gzhelian; Stephanian C–lower Autunian) with a new locality Štikov—as well as a full revision of historical collections housed in Czech institutions. The tetrapod ichnofossil record now includes six ichnotaxa: *Amphisauropus*, *Batrachichnus*, *Dimetropus*, *Dromopus*, *Ichniotherium*, and *Limnopus.* Special attention is given to the palaeoecological assessment of track localities and spatiotemporal distribution of track producers in the KPB. Our study provides unique evidence of terrestrial life during the latest Pennsylvanian, associated with a lakeshore environment. This, in turn, enriches our understanding of the diversity of terrestrial palaeoecosystems within the intra-Variscan basins of equatorial Pangaea.

## Geological setting

**Figure 1:**
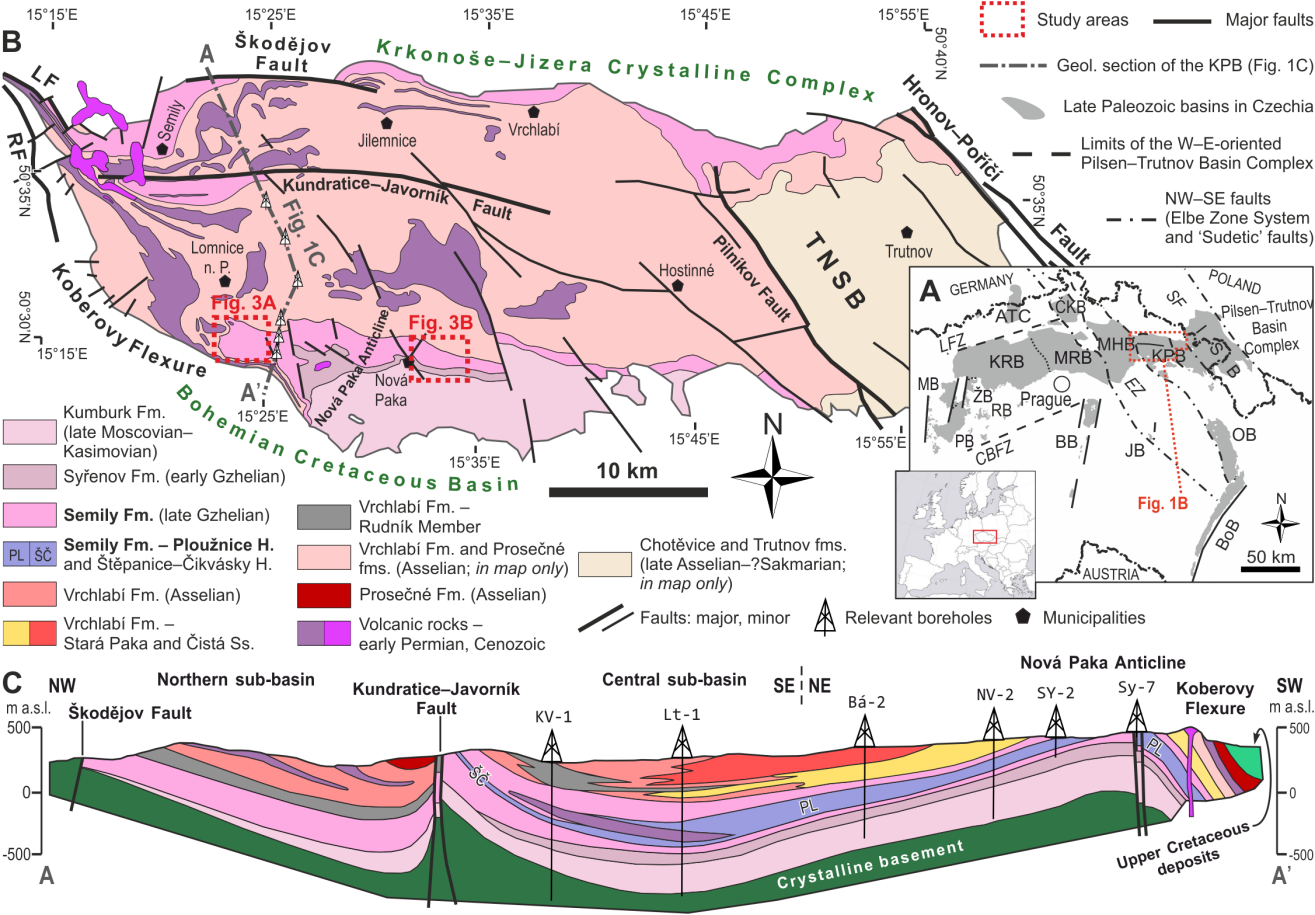
Location of the studied area. (A) Present-day perspective of the Late Paleozoic continental basins of the Bohemian Massif (Opluštil et al., 2013, amended). Abbreviations: ATC, Altenberg–Teplice Caldera; BB, Blanice Graben; BoB, Boskovice Graben; CBFZ, Central Bohemian Fault Zone; ČKB, Česká Kamenice Basin; EZ, Elbe Zone; ISB, Intra-Sudetic Basin; JB, Jihlava Graben; KPB, Krkonoše Piedmont Basin; KRB, Kladno–Rakovník Basin; LFZ, Litoměřice Fault Zone; MB, Manětín Basin; MHB, Mnichovo Hradiště Basin; MRB, Mšeno–Roudnice Basin; OB, Orlice Basin; PB, Pilsen Basin; RB, Radnice Basin; SF, Sudetic faults; ŽB, Žihle Basin. (B) A detailed map of the KPB (compiled after Martínek et al., 2006; Stárková et al., 2010; Stárková et al., 2017; and Prouza, Coubal & Adamovič, 2013). Study areas indicated. Abbreviations: LF, Lusatian Fault; RF, Rovensko Fault; TNSB, Trutnov–Náchod sub-basin. (C) Geological section of the western part of the KPB (amended after V. Prouza in Pešek et al., 2001; Stárková et al., 2010). Location of the section indicated in Fig. 1B.

**Figure 2:**
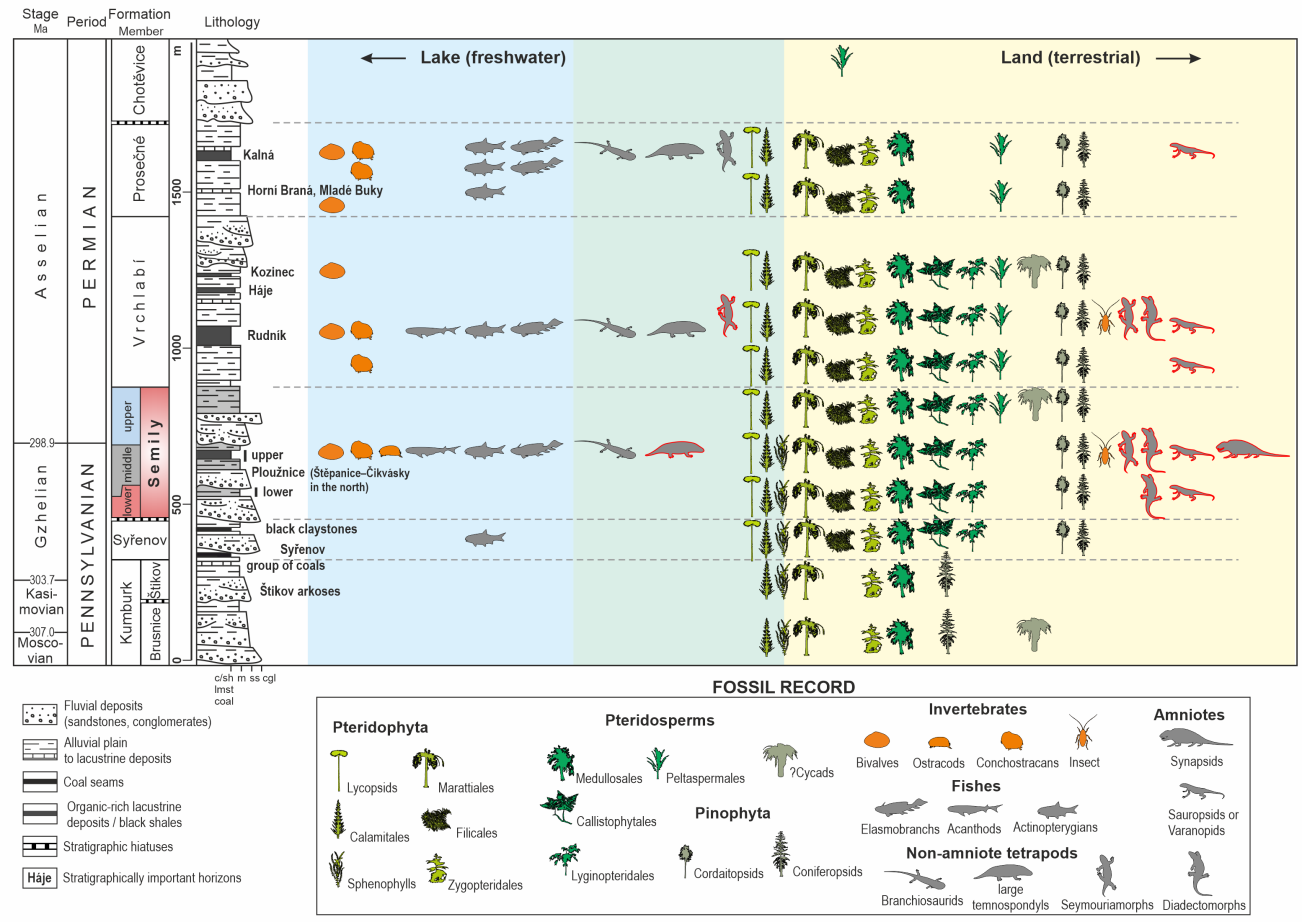
Interpretation of palaeodiversity in the Krkonoše Piedmont Basin based on the area’s fossil record. Taxa highlighted in red represent those known only from ichnological evidence. Stratigraphy follows Opluštil et al. (2016b). The lithological column is modified after Stárková et al. (2015). Fossil record data are compiled from Fritsch (1895), Fritsch (1901), Frič (1912a), Frič (1912b), Kamarád (1951), Kamarád (1959), Rieger (1958), Rieger (1968), Pešek et al. (2001), Zajíc (2007), Zajíc (2014), Štamberg & Zajíc (2008), Opluštil et al. (2016a), Opluštil et al. (2016b), Opluštil, Šimůnek & Mencl (2022), Mencl et al. (2013), Schneider et al. (2020), and Štamberg & Lapacík (2018) and Štamberg (2023).

The Krkonoše Piedmont Basin (KPB) is a ∼1,100 km^2^ large W–E elongated basin that is part of an extensive Pennsylvanian–Permian Bohemian basin system (Fig. 1), also termed as the Pilsen–Trutnov Basin Complex (PTBC–Cháb et al., 2010; Opluštil et al., 2016b). Palaeogeographically, the PTBC was located within the tropical belt, between ca. 2° and 4° north of the palaeoequator (Krs & Pruner, 1995: Fig. 2). The KPB is located in northeastern Bohemia in the southern foothills of the Krkonoše Mountains (Fig. 1B) and is formed by several separate sub-basins altogether deformed into a set of synclinoria and anticlinoria (Fig. 1C). The infill of the PTBC contains up to ∼1,400 m (cf. Pešek et al., 2001) of the Middle Moscovian (lower Bolsovian) to the lower Permian (Asselian; cf. Opluštil et al., 2016b) strata predominantly red, coal- and fossil- poor formations: Kumburk, Semily (except for its middle part), Vrchlabí, Prosečné and Chotěvice formations (Fig. 2). Compared to the central and western part of the PTBC, the basin fill of the KPB contains substantially less of grey, coal-bearing deposits. Coal seams are preserved within the Syřenov Fm., and within the middle part of the Semily Fm. Several unconformities in the basin fill (Fig. 2) record ∼1–4 Myr hiatuses reflecting late orogenic to intraplate tectonic processes that resulted in reactivation of Variscan faults, inversion of older basin fill and rearrangement of basin geometry (Opluštil et al., 2016b; Nádaskay et al., 2024; Nádaskay et al., 2025). Therefore, the infill of the KPB records several tectonosedimentary cycles (Nádaskay, 2021). The Semily Fm., the focus of this study, represents the basal part of the Late Pennsylvanian—early Permian tectonosedimentary cycle, generated by widespread extension within the Variscan Belt and locally affected by transtensional tectonics (Nádaskay et al., 2024; Nádaskay et al., 2025). Based on the rich fossil plant assemblages from individual stratigraphic levels of the KPB, an increasing seasonality is traceable from the Pennsylvanian towards the Permian (Opluštil, Šimůnek & Mencl, 2022).

### Semily formation with focus on the Ploužnice Horizon

The Semily Fm. represents a ∼500 m thick sequence of predominantly reddish conglomerates, sandstones, siltstones and claystones (Pešek et al., 2001; Opluštil et al., 2016b). In its typical development, the formation is divided into three parts—lower, middle and upper (Fig. 2; cf. Pešek et al., 2001), each with distinct lithofacies development. The lower part of the formation is dominated by coarse clastic deposits that are particularly concentrated along the northern, tectonically-driven basin margin. These are represented by up to ca. 120 m thick (cf. Pešek et al., 2001) often poorly-sorted coarse-grained conglomerates or even breccias containing mostly phyllite and quartz clasts from the nearby Krkonoše–Jizera Crystalline Complex (Fig. 1B). In the south, the basal part of the Semily Fm. is formed by conglomerates up to ∼40 m thick (cf. Pešek et al., 2001) with good sorting and clast rounding and coarse-grained sandstones.

The middle part of the formation is dominated by fine-grained deposits and is ∼130 m thick in the south and ∼100 m thick in the north. In the south, the middle part of the formation contains a succession formed by varicoloured siltstones, claystones with subordinate limestones, and contains distinctive lenses of cherts, interpreted as lacustrine deposits grouped into the Ploužnice Horizon. This unit is formed by two lacustrine intervals (lower and upper Ploužnice Horizons), each ∼10–60 m thick, separated by alluvial deposits—brownish-reddish interval of predominantly mudstones and fine-grained sandstones, ∼10–30 m thick (Pešek et al., 2001). The lacustrine deposits are also represented by the Štepanice–Čikvásky Horizon, a presumed continuation of the Ploužnice Horizon to the northern part of the Central sub-basin (Fig. 1C). Both units are traditionally termed as ‘horizons’, although their greater thickness and varied lithology contradict the concept of ‘stratigraphic horizon’ as a thin marker of distinctive lithology (cf. Courel et al., 2008), and should be defined as a member of the Semily Fm.

The Ploužnice Horizon reportedly contains numerous flora remains of lycopsids, Equisetids (Calamites, Sphenophylls), Ferns (Marattiales, Zygopteridales, Filicales), Pteridosperms (Medullosales, Lyginopteridales, Callistophytales, Peltaspermales) and Gymnosperms (Cordaitopsids, Coniferopsids) as well as a rich fauna of bivalves, ostracodes, conchostracans, blattoid insect, arachnids, elasmobranchs, actinopterygians, and tetrapod footprints (Fig. 2; Fritsch, 1901; Frič, 1912a; Frič, 1912b; Kamarád, 1951; Kamarád, 1959; Rieger, 1958; Rieger, 1968; Pešek et al., 2001; Zajíc, 2007; Štamberg, 2001; Štamberg & Lapacík, 2018; Štamberg, 2023; Štamberg, 2024; Štamberg & Zajíc, 2008; Mencl et al., 2013; Opluštil et al., 2016a; Opluštil et al., 2016b; Opluštil, Šimůnek & Mencl, 2022; Schneider et al., 2020).

**Figure 3:**
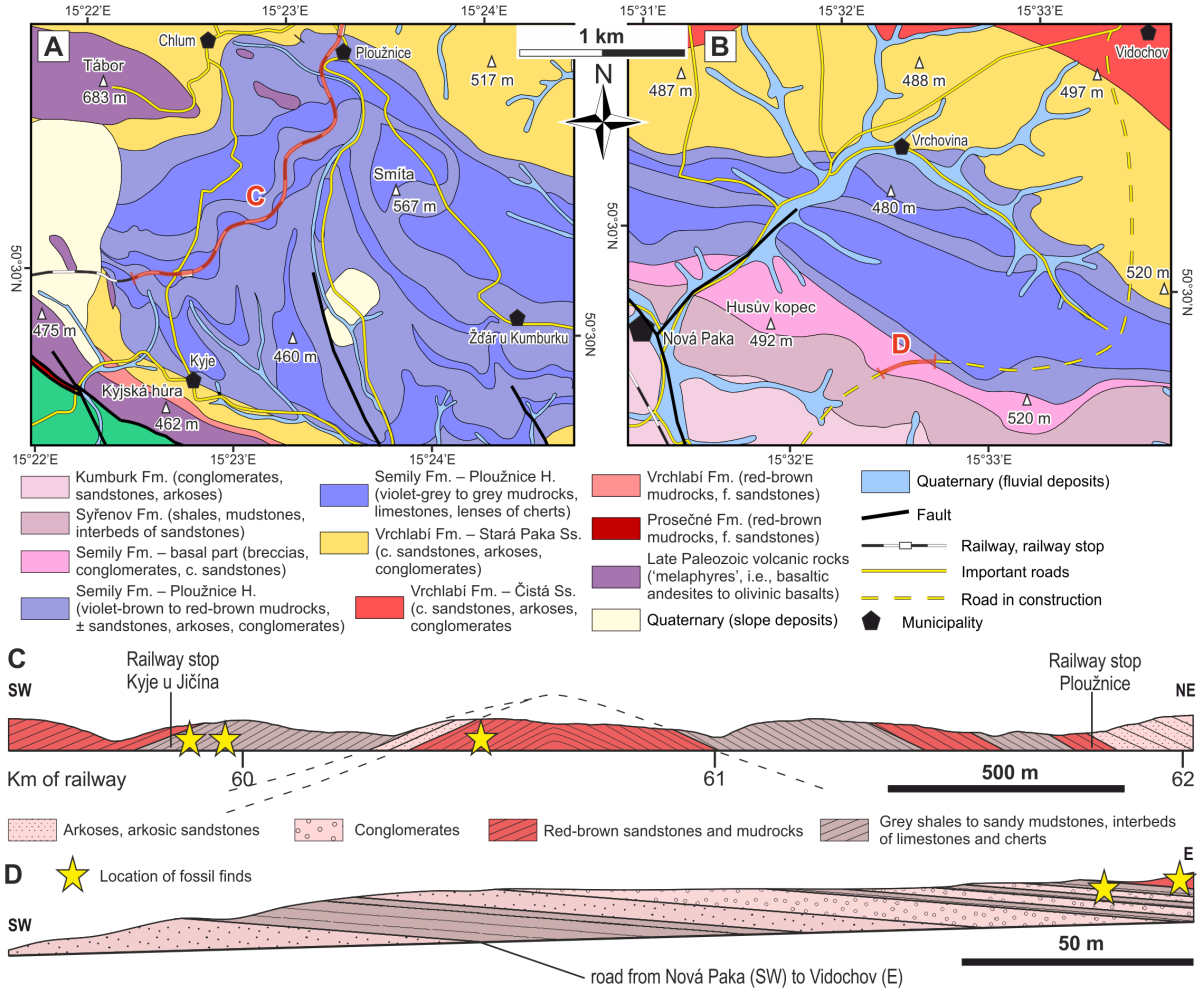
Geological conditions of the studied area. (A) A geological map of the vicinity of the studied locality Kyje–Ploužnice railway cut. Modified after Stárková et al. (2013). (B) A geological map of the vicinity of the studied locality Štikov—a roadcut at a road construction site east of Nová Paka. Modified after Stárková et al. (2017). (C) The entire railway cut, showing the lithological variability and tectonic deformation of the Ploužnice Horizon, was first depicted by Purkyně (1929). (D) The schematic lithological profile of Štikov—roadcut under the Ploužnice Horizon.

The Late Pennsylvanian fossil fauna of the KPB has been known since the beginning of the 20th century based on the discoveries in the railway cut between Ploužnice and Kyje (Figs. 3A, 3C) and the Krsmol locality (Fritsch, 1901; Frič, 1912a; Frič, 1912b) representing the localities of the Ploužnice Horizon (Stephanian C). Above the Ploužnice Horizon, alluvial-lacustrine deposits of the upper part of the Semily Fm. pass into the Vrchlabí Fm. whose basal part of is lithologically similar but devoid of cherts and features monotonous colours (brownish/reddish and/or greyish). This interval contains the Carboniferous-Permian boundary (Opluštil et al., 2013; Opluštil, Šimůnek & Mencl, 2022). Based on the litho- and biostratigraphic correlation of the Ploužnice Horizon with the Klobuky Horizon (Líně Fm.) in central Bohemia, dated at 298.97 ± 0.09 Ma (Opluštil et al., 2016a), and unpublished dating of the base of the Vrchlabí Fm. at 298.72 Ma (cf. Nádaskay et al., 2024), the uppermost part of the Semily Fm. is likely early Permian in age.

**Figure 4:**
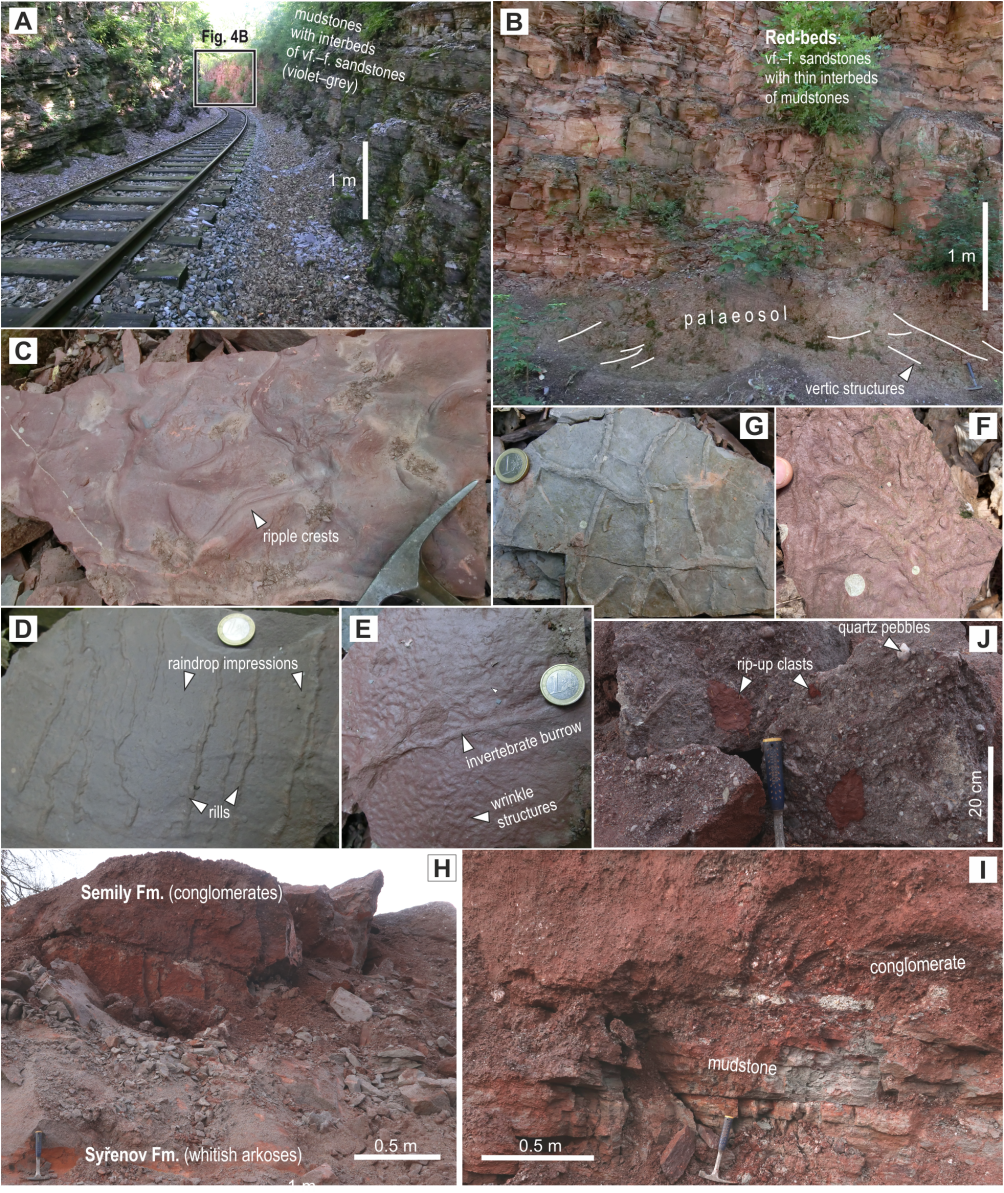
Photographs of the studied localities. (A) The western end of the Kyje–Ploužnice railway cut exposing predominantly violet–grey-coloured mudstones with interbeds of very fine- to fine-grained sandstones, occasionally containing volcaniclastic layers and cherts. The cut is exposed on both sides of the railway, with walls reaching up to 10 m in height. Red-beds are visible in the background. (B) Red-beds in the middle part of the Kyje–Ploužnice railway cut. The observed palaeosol containing abundant carbonate nodules and conspicuous vertic structures, is overlain by a succession of reddish–brownish very fine- to fine-grained sandstones with thin interbeds of mudstones. (C) A slab of reddish sandstone with ripples in three-dimensional relief, covered by a thin mud drape. Kyje–railway cut, middle part of the section (red-beds). (D) An example of negative raindrop impressions accompanied by rills produced by surface runoff. These features indicate that the mudflat was subaerially exposed at the time. Kyje–railway cut, middle part of the section (red-beds). (E) Irregular structures, possibly a form of wrinkle structures (*cf.* Porada & Bouougri, 2007), which may have formed as a result of bacterial colonisation of the sediment surface in shallow-water pool on the mudflat. Kyje–railway cut, middle part of the section (red-beds). (F) Fine-grained sandstone with abundant and well-defined invertebrate burrows. Kyje–railway cut, middle part of the section (red-beds). (G) An example of mud cracks (filled with fine sand) preserved in a slab of pale grey mudstone from the upper part of the section Kyje–railway cut. (H) Conglomerates from the basal part of the Semily Formation. The conglomerates are clast-supported, relatively poorly sorted, and contain rounded clasts composed predominantly of vein quartz and micaschists. Štikov–roadcut. (I) A boundary between Syřenov and Semily formations at the Štikov–roadcut. The Syřenov Formation is represented here by whitish arkoses overlain by incised reddish conglomerates (Fig. 4H) of the Semily Formation. Note the bed inclination in conglomerates. (J) Upper part of the section at the locality Štikov–roadcut, displaying grey mudstones overlain by reddish conglomerate.

### Kyje–Ploužnice railway cut (Ploužnice Horizon)

**Figure 5:**
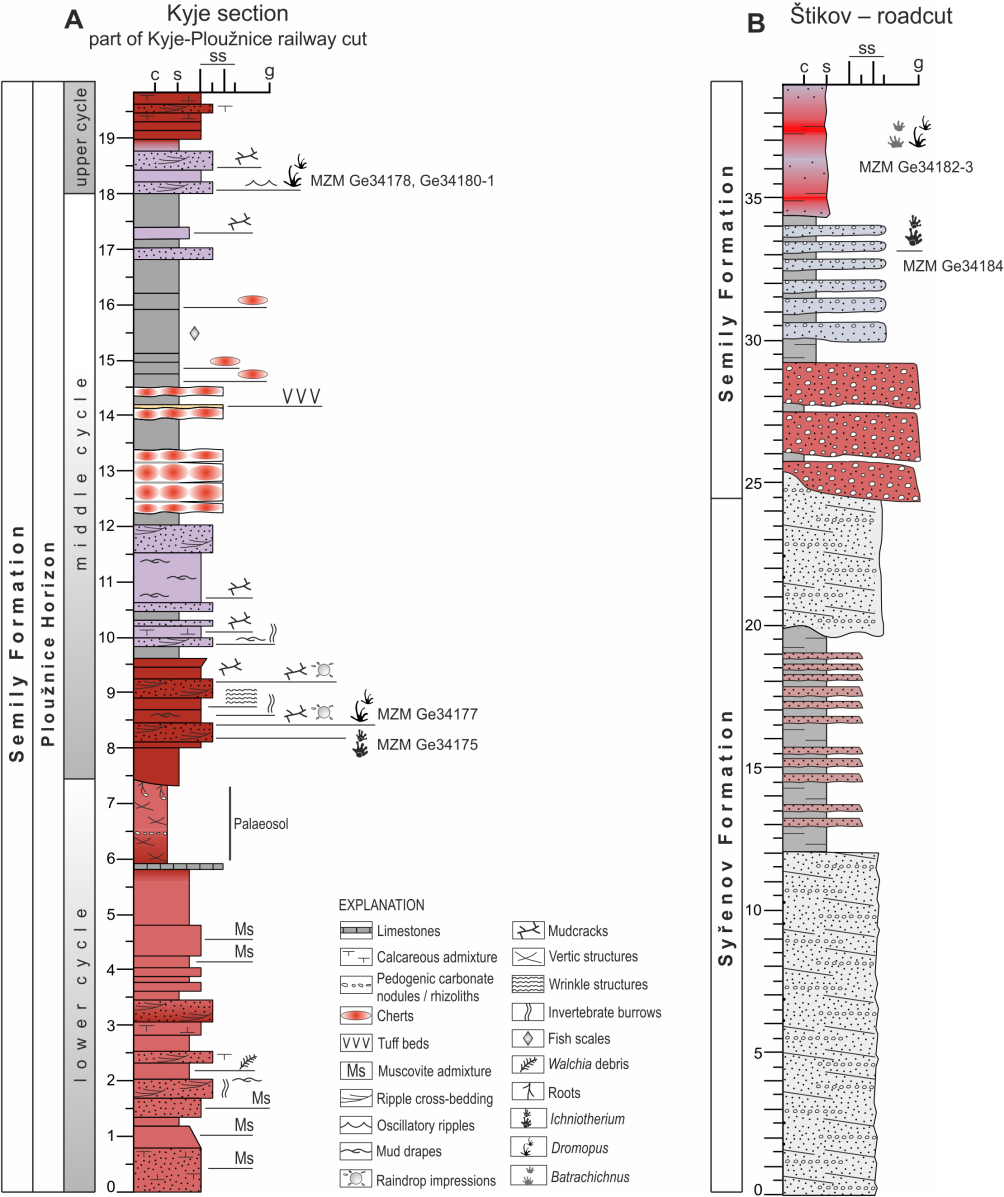
Sedimentary logs of studied localities. (A) Kyje section; (B) Štikov–roadcut. The positions of tetrapod footprints excavated in 2024 and 2025 are indicated. Section thickness in meters.

Palaeontological material has been documented from this section of the Kyje–Ploužnice railway cut since the early 20th century (Frič, 1912a; Frič, 1912b), making it one of the historically significant fossil sites in the region. The railway cut created several outcrops in the Ploužnice Horizon between Kyje and Ploužnice railway stops (Fig. 3A) and was already described in detail by Frič (1912a: figs. 12, 13; 1912b: figs. 12, 13) and Purkyně (1929; Fig. 3C). The series of railway cuts was studied sedimentologically by Blecha et al. (1997). Among the several exposures described by these authors, this paper focuses on the section closest to the Kyje railway stop, where the Ploužnice Horizon is well exposed on both sides of the railway (Fig. 4A). The Kyje section (Fig. 5A) displays two fining-upward cycles. The lower cycle is formed by mudstones at the base, overlain by fine-grained sandstones with ripple cross-bedding (Fig. 4B) or low-angle cross-bedding, often with sharp bases and sometimes with conspicuous channelization (Fig. 4B). The alternation of mudstones and sandstones is topped by a silty-sandy limestone, which is overlain by a pedogenic horizon (Fig. 4B) characterised by distinct vertic slickensides and abundant carbonate nodules. The lower cycle (Fig. 5) is predominantly brown- or red-brown coloured. The middle cycle (Fig. 5) starts at the base with very fine to fine-grained sandstones, often silty or argillaceous, with mud drapes or even thin interbeds of mudstones (Fig. 4C). Mud drapes are occasionally associated with rain rills and rain-drop impressions (Fig. 4D), sometimes with wrinkle structures (Fig. 4E) possibly left by microbial mats and with invertebrate burrows (Fig. 4F). Mudcracks (Fig. 4G) are present both in red- and violet/grey-coloured deposits.

These sandstones pass upward into ∼7–8 m thick sequence of predominantly violet and grey mudstones with cm to first dm thick interbeds of greyish sandstones (Fig. 4A). The upper part of this sequence is apparently finer, mudstone-dominated, and contains nodular interbeds of reddish cherts and ∼1 cm thick volcaniclastic layers. The top of the section, inclined towards the Kyje railway stop, is represented by alternation of violet to brownish mudstones and brownish sandstones. These deposits represent a lower part of an upper, incompletely exposed cycle.

Concerning fossil finds, the flora is represented by lycopsids (*Asolanus camptotaenia*, *Halonia* (*Ulodendron*)*, Stigmaria ficoides, Lepidostrobus variablis, Lepidophyllum* sp.), equisetales (*Sphenophyllum oblongifolium, Asterophyllites equisetiformis, Annularia spinulosa*, *Calamites gigas, C. cruciatus, C. undulates*, *C. suckowii*, *C. cistii, Calamostachys tuberculata etc.*), ferns (*Cyathocarpus arboreus, Pecopteris arborescens*, *Scolecopteris cyathea*, *Acitheca polymorpha etc.*), pteridosperms (*Odontopteris subcrenulata, O. schlotheimii, Callipteridium pteridium, Ca. costei, Neuropteris zeilleri, N. cordata , Neurodontopteris auriculata etc.*) and gymnosperms (*Culmitzschia* cf. *speciosa, Walchia piniformis, Ernestiodendron filiciforme*, *Cordaites borassifolius*) (Purkyně, 1929; Němejs, 1932; Štamberg & Lapacík, 2018; Šimůnek, Lapacík & Mencl, 2022; Opluštil, Šimůnek & Mencl, 2022). Fossil fauna comes from thin layer (∼0.5 m) of dark red claystone originally called “bonebeds” by Frič (1912a) and Frič (1912b) and from the lower part of varicoloured tuffitic claystones (Štamberg, 2023). Fossils are represented by wings of the blattoid insect *Spiloblattina lawrenceana*, *Sysciophlebia rubida*, *Neorthroblattina* cf. *multineuria* and *Anthracoblattina* sp., scales of elasmobranchs Xenacanthidae and *Sphenacanthus* sp., scales and fin spines of *Acanthodes* sp., scales and bones of actinopterygians *Elonichthys* sp*., Sphaerolepis kounoviensis*, *Spinarichthys disperses, Progyrolepis speciosus* and *Zaborichthys fragmentalis* and bones of branchiosaurid (Frič, 1912a; Frič, 1912b; Schneider, 1983; Zajíc, 2007; Štamberg & Lapacík, 2018; Štamberg, 2023). As mentioned above, Frič (1912a) and Frič (1912b) described a tetrapod ichnotaxon *Saurichnites calcaratus*=*Dromopus lacertoides* (Geinitz, 1861) in a “dark” shale (the layer corresponds to a red mudstone containing ichnofossils of *Dromopus*; *e.g.*, MZM Ge34179) in an outcrop near the railway milestone marking the distance 60.5 km. Later Haubold & Katzung (1975) expanded the list of ichnotaxa from the Kyje–Ploužnice railway cut with *Anthichnium salamandroides*=*Batrachichnis salamandroides* (Geinitz, 1861), *Amphisauropus latus*=*Amphisauropus kablikae* (Geinitz & Deichmüller, 1882), *Dromopus lacertoides* and *Ichniotherium cottae,* which are revised in this study and described and depicted for the first time.

### Krsmol—locality “Hluboká rokle” (Ploužnice Horizon)

The locality Krsmol is well known from the beginning of the palaeontological research in the KPB (Fritsch, 1901; Frič, 1912a; Frič, 1912b). The outcrop is located in the upper part of a deep erosional gorge. Its current condition is described in detail by Štamberg (2024). From the base of profile, he described 30 cm thick chert, five cm thick massive red sandstone, well bedded 10 cm thick grey siltstone, with fauna on the base and 100 cm thick purplish siltstone with fauna. According to Štamberg (2024), these layers are comparable to the “bonebed” at the Kyje–Ploužnice railway cut. Flora is represented by lycopsids (*Sigillaria brardi, Lepidodendron* sp. *etc.*), equisetales *Calamites gigas* and pteridosperms *Odontopteris schlotheimii, Neurodontopteris auriculata* and *Callipteridium pteridium* (Purkyně, 1929; Němejs, 1932; Opluštil, Zajíc & Svoboda, 2022). The fauna is represented by bivalves *Carbonicola bohemica*, arachnids *Anthracolysa* sp., wings of blattoid insect *Sysciophlebia rubida*, fin spines, scales, teeth, and fin spin of elasmobranchs *Turnovichthys magnus*, *Lissodus* sp., *Orthacanthus* sp. and *Bohemiacanthus* sp., scales of acanthodians and scales and teeth of the actinopterygians *Elonichthys* sp*.* and *Sphaerolepis kounoviensis* (Fritsch, 1901; Frič, 1912a; Frič, 1912b; Štamberg, 2001; Štamberg, 2024; Zajíc, 2007).

### Štikov—roadcut

This locality featured a temporary outcrop (*e.g.*, Fig. 4H) created during the construction of a new road bypassing the town of Nová Paka (Fig. 3B), which was exposed during the years 2023–2024. The approximately 700 m long outcrop exposes a NE dipping sequence (Fig. 3D) of conglomerates and sandstones alternating with siltstones (ca. 5 m thick; Fig. 4I) that forms the lower part of the Semily Fm., *i.e.,* beneath the Ploužnice Horizon (Fig. 5B). This sequence is underlain by whitish arkoses (Fig. 4H) and contains grey mudstones with thin interbeds of violet-grey sandstones, which belong to the Syřenov Fm. (Fig. 5B). Upsection, the conglomerates of the basal part of the Semily Fm., typically clast-supported, poorly-sorted with pebbles of quartz and micaschists (Fig. 4J) pass into alternating sandstones and siltstones (∼5 m thick), and the exposed part of the sequence ends with red mudstones. In the upward direction, a colour of the sediments changes from grey-violet to red. The fossil tracks of *Ichniotherium* come from the sequence of conglomerates alternating with ∼30 cm thick siltstones interbeds containing *Dromopus* isp. (MZM Ge34182), cf. *Batrachichnus* (MZM Ge34182) and cf. *Ichniotherium* (MZM Ge34183) footprints (M Stárková, pers. comm., 2023). This sequence is overlain by predominantly red mudstones.

## Materials & Methods

**Figure 6:**
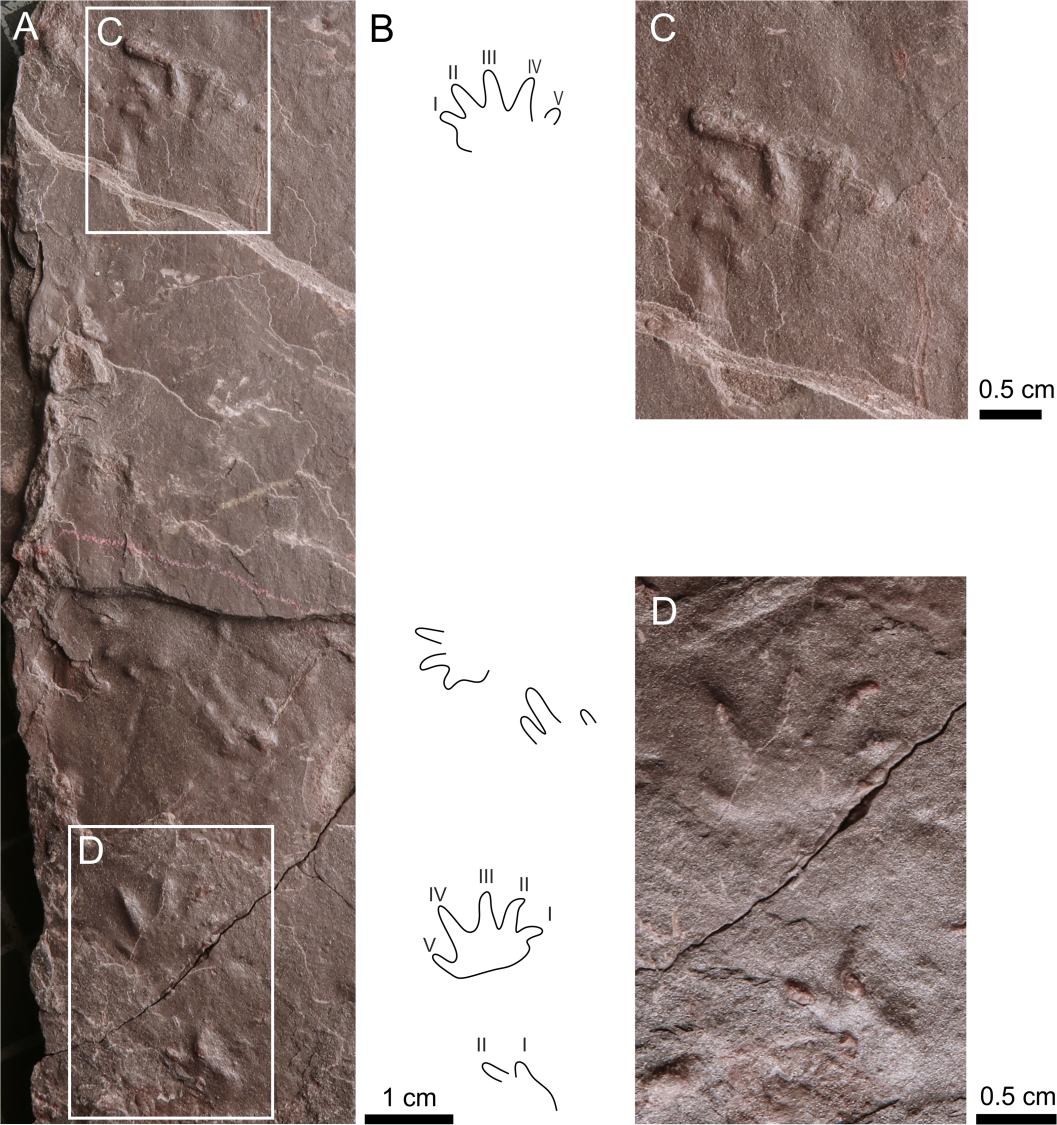
*Amphisauropus kablikae*. (A) CGS JZ626, incomplete trackway in convex hyporelief, (B) Outline drawing of the trackway, (C) magnified manus imprint, (D) and manus imprint accompanied by incompletely preserved pes imprint.

**Figure 7:**
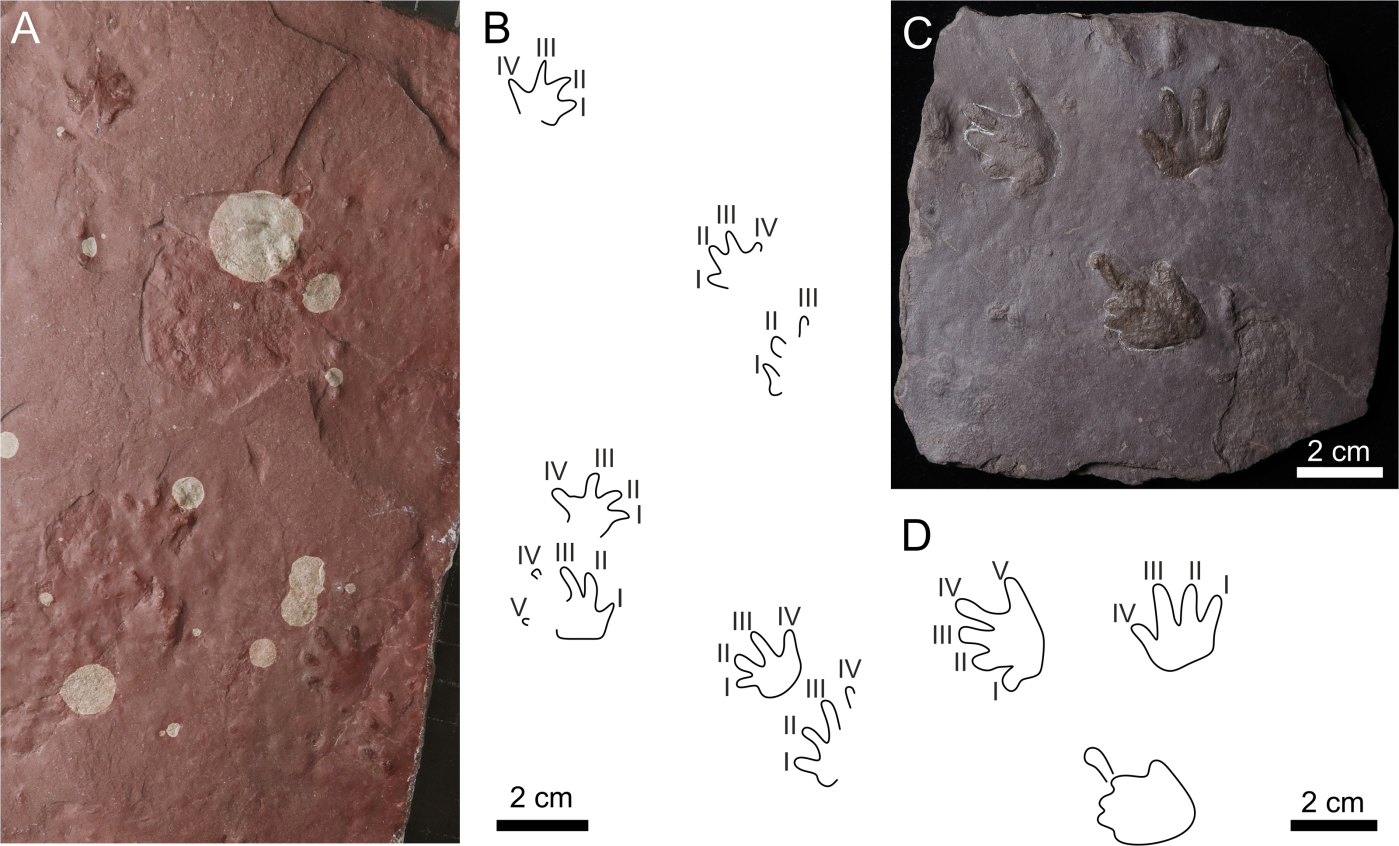
*Batrachichnus salamandroides*. (A) CGS XA738, trackway in convex hyporelief; (B) outline drawing of CGS XA738; (C) MZM Ge31124, isolated three tracks in convex hyporelief, (D) outline drawing of MZM Ge31124.

**Figure 8:**
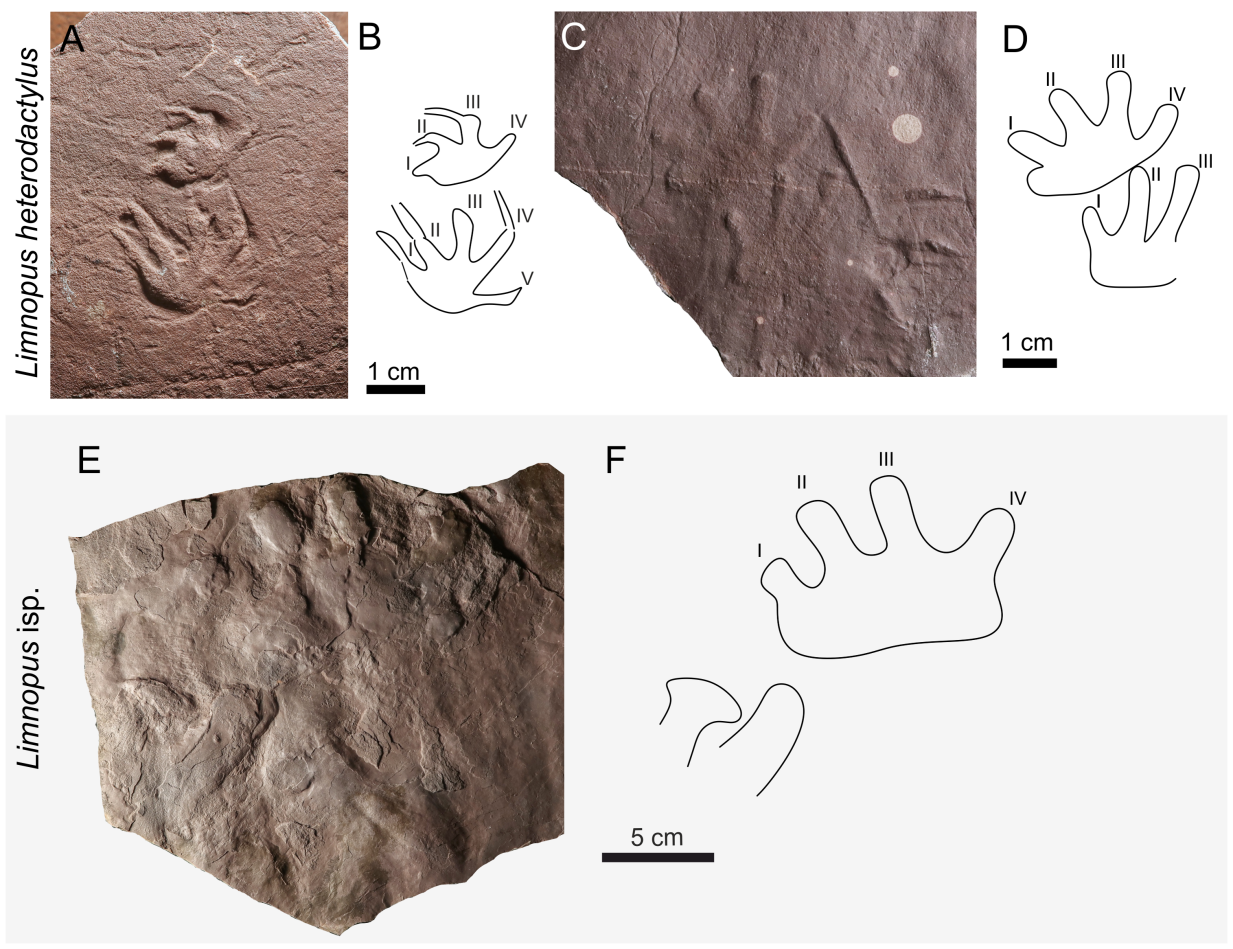
*Limnopus heterodactylus* (A–D) and *Limnopus* isp. (E–F). (A) NPM P3573, manus pes couple in convex hyporelief, (B) outline drawing of NPM P3573; (C) CGS XA741, manus-pes couple in convex hyporelief; (D) outline drawing of CGS XA741; (E) MZM Ge34174, incomplete manus-pes couple in convex hyporelief; (F) outline drawing of MZM Ge34174.

**Figure 9:**
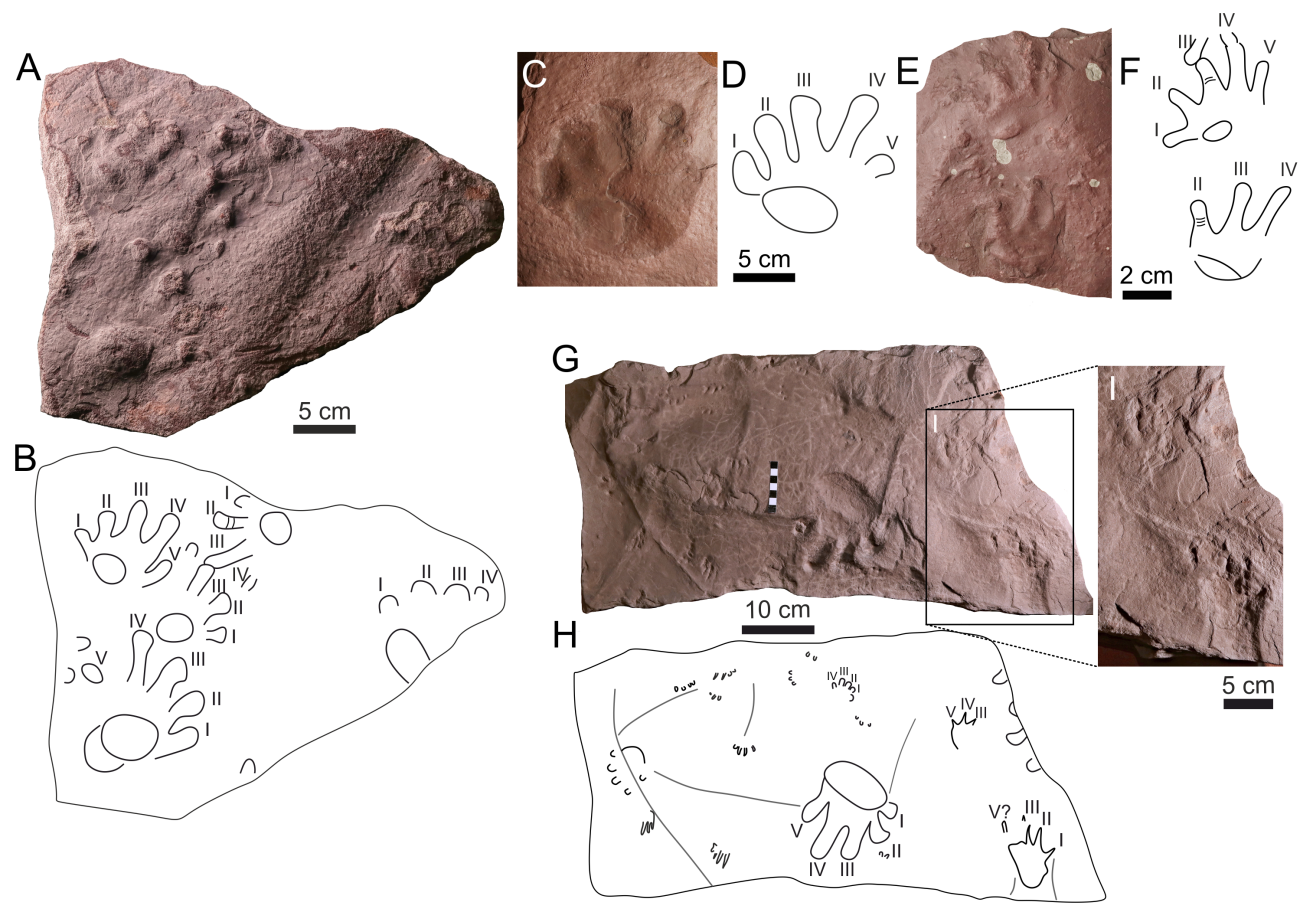
*Ichniotherium cottae* (A–H) and *Dimetropus* isp. (I). (A) MZM Ge34184, manus pes couple with two isolated tracks in convex hyporelief; (B) outline drawing of MZM Ge34184; (C) NPM P103, isolated pes imprint in concave epirelief; (D) outline drawing of NPM P103; (E) CGS XA737, manus-pes couple in convex hyporelief; (F) outline drawing of CGS XA737; (G) NPM P307, manus imprint in concave epirelief; (H) outline drawing of NPM P307, (I) NPM P307, manus-pes couple in concave epirelief.

**Figure 10:**
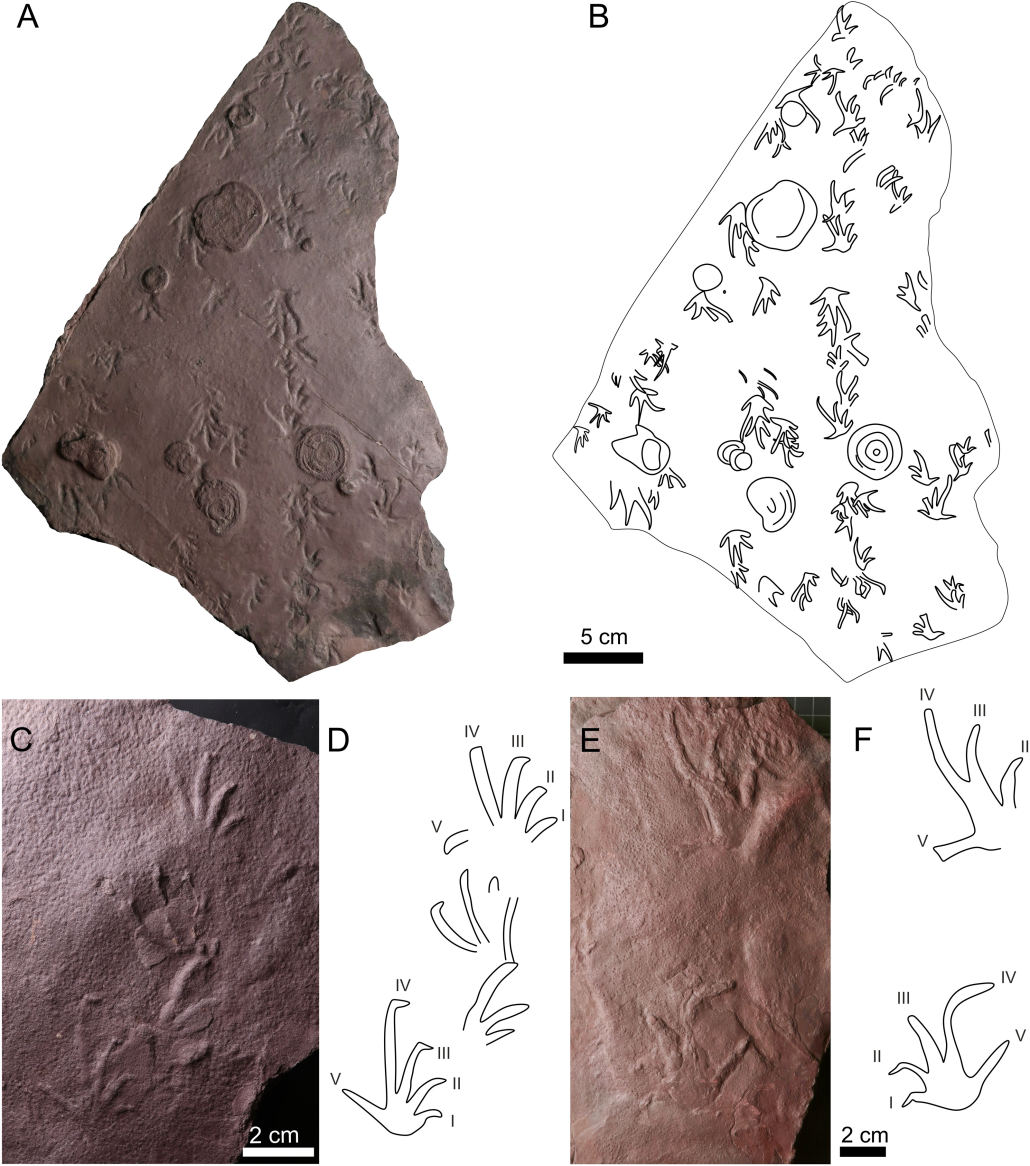
*Dromopus lacertoides*. (A) NM M4949a, several trackways in concave epirelief; (B) outline drawing of NM M4949a; (C) MZM Ge34176, manus-pes couple and two isolated tracks in convex hyporelief; (D) outline drawing of MZM Ge34176; (E) GGS XA742, two tracks in convex hyporelief (F) outline drawing of CGS XA742.

The study is based on tetrapod track specimens from several localities within the Semily Fm., Krkonoše Piedmont Basin: Kyje (a section that is part of Kyje–Ploužnice railway cut) Krsmol, and Štikov roadcut (Figs. 6–10). The tracks are preserved as both concave epirelief and convex hyporelief, and consist of isolated tracks, manus-pes couples, and trackways. The specimens are housed at the Czech Geological Survey in Prague (CGS), the Moravian Museum in Brno (MZM), the National Museum in Prague (NM), and the Nová Paka City Museum (NPM).

The stratigraphically oldest specimen (Figs. 9A–9B) MZM Ge34184 (convex hyporelief) includes a manus–pes couple and three isolated tracks that were discovered in 2024 by the authors (GC, JB) in the excavated material from the temporary outcrop that was created during the construction of the bypass road from Nová Paka to Vidochov (Fig. 3B). The outcrop was buried during subsequent construction in 2025 and no longer exists. Due to limited access, the sedimentary profile at this location could not be thoroughly studied, and is therefore simplified without a detailed sedimentological investigation (Fig. 5B). The specimen MZM Ge34184 is preserved in violet sandy conglomerate. The conglomerate layers are located in the lower part of the Semily Fm. (Stephanian C).

In 2024 and 2025, the authors (GC, JB, RN) discovered track specimens in the Kyje–Ploužnice railway cut including MZM Ge34174–Ge34181, most of which were excavated directly from the reddish and purplish coloured layers and marked in the profile (Fig. 5A). From the same outcrops also come the specimens CGS JZ626 and JZ632 discovered in the 1990s by Z. Šimůnek (ČGS), and the specimens CGS XA736, XA737, XA738, XA739, XA740, XA741, XA742, XA743, XA744, XA746 discovered by K. Havlata most probably in the 1930s. The Havlata’s collection was most likely mentioned in the study by Haubold & Katzung (1975), although their ichnotaxa list is not accompanied by any description or catalog numbers. The historical collections of track specimens from the Kyje–Ploužnice railway cut always lack precise localization within the profile and probably was collected from the talus. However, their preservation in fine sandstone to claystone with reddish to purplish hues suggests the presence of at least two distinct fossiliferous layers.

Some collections from the 20th century housed in the Czech museums marked separately under the localities Kyje (MZM Ge 31124, NPM P103, NPM P104) and Ploužnice (NPM P307, NM M4949a) most likely come from the same railway cut, which is well known and often cited from the beginning of palaeontological research in this basin (see Fritsch, 1901; Frič, 1912a; Frič, 1912b). Specimens NPM P103, NPM P104 and NPM P307 were donated to the Nová Paka City Museum by V. Fejfar in 1926 in the first case and by K. Tuček in 1971 in the other cases. NM M4949a housed at the National Museum Prague was likely donated at the turn of the 19th and 20th centuries.

The specimen NPM P3573 from the locality Krsmol was acquired by the Nová Paka City Museum from K. Tuček in the 1970s.

The anatomical terminology used to describe fossil footprints follows Leonardi (1987) and Lallensack, Leonardi & Falkingham (2025). The specimens bearing fossil tracks were measured with a digital calliper on both convex hyporeliefs and concave epireliefs and photographed with a Canon PowerShot G7 camera. Some of the specimens collected in the field (MZM Ge34174, MZM Ge34176, MZM Ge34184) were cleaned and subsequently remeasured from photographs using ImageJ software.

## Results

### Systematic palaeoichnology

**Table:** 

***Amphisauropus,*** **Haubold, 1970**
***Amphisauropus kablikae*** **(Figs. 6A–6D)**

**Referred material**. CGS JZ626—convex hyporelief; CGS JZ632—concave epirelief. The specimens are convex hyporelief and concave epirelief of the same original sample.

**Description:**

The manus imprints are pentadactyl and plantigrade whereas the pes imprints are incomplete semidigitigrade to digitigrade. The manus imprints are about 9 mm long and about 11.5 mm wide (Table S1). The palm impressions show a rectangular shape. The length of the manual digit impressions gradually increases from I to IV. Digit V is shorter than digit II. The imprints of manual digits II–III are distally bent inward. Manual digits I have the typical basal pad. The digits show round terminations. The manus imprint is turned inward compared to the pes imprint. The trackway shows an alternating arrangement of successive manus and pes imprints. There is no overprinting within the manus-pes couple.

**Remarks:**

The specimens described above show the main diagnostic features of *Amphisauropus kablikae*, including pentadactyl wide manus imprint with relatively thick digits with rounded terminations, the longest manual digit IV is similar in size as digit III, relatively short manual digit V imprints, rectangular palm impressions, and a strongly inwards oriented manus imprint in relation to the pes imprint (*e.g.*, Voigt, 2005; Voigt et al., 2012; Marchetti et al., 2015a).

*Amphisauropus* tracks have been known from the Pennsylvanian to the Cisuralian (early Permian), while the vast majority of specimens have been discovered in the Cisuralian of Europe (*e.g.*, Fritsch, 1901; Pabst, 1905; Haubold, 1971; Haubold, 1996; Voigt, 2005; Voigt, 2015; Gand & Durand, 2006; Avanzini et al., 2008; Voigt et al., 2012; Marchetti, Avanzini & Conti, 2013; Marchetti et al., 2015a; Marchetti et al., 2015b; Marchetti, Mujal & Bernardi, 2016; Marchetti, Voigt & Santi, 2018; Mujal et al., 2016; Santi et al., 2020; Calábková, Březina & Madzia, 2022), North America (Haubold et al., 1995; Lucas, Lerner & Haubold, 2001; Lucas, Spielmann & Lerner, 2009; Van Allen, Calder & Hunt, 2005; Voigt & Lucas, 2015a; Voigt & Lucas, 2015b; Voigt & Lucas, 2016; Voigt & Lucas, 2017; Marchetti et al., 2019; Marchetti et al., 2020a), and North Africa (Voigt et al., 2011a). The *Amphisauropus* tracks from the Ploužnice Horizon represent indisputable evidence of the occurrence of this ichnotaxon in the Gzhelian. The older occurrence of *Amphisauropus* was designated by Lucas & Stimson (2025) and Marchetti et al. (2021), which comes from the Keota Sandstone Member of Middle Pennsylvanian in Oklahoma.

**Table:** 

***Batrachichnus*** ** Woodworth, 1900**
***Batrachichnus salamandroides*** **(Geinitz, 1861) (Figs. 7A–7D)**

**Referred material**. CGS XA738—convex hyporelief, trackway consists of four manus-pes couples; MZM Ge31124—convex hyporelief, isolated three tracks.

**Description:**

The manus imprints are tetradactyl and plantigrade, and the pes imprints are pentadactyl and plantigrade to digitigrade. The footprints are up to ∼27 mm long. The manus imprints are about 1/5 smaller than the pes imprints. The manus imprints are slightly wider than long, whereas the pes imprints are almost as long as wide (Table S1). The manual digits imprints are relatively short with rounded terminations. The length of the manual digits increases from I to III, and digit IV is about as long as the digits I or II. The length of the pedal digits increases from I to IV, and V is shorter than III. The manual digit I often has a distinctly impressed basal pad. The palm and sole impressions are most often wider than long. The manus imprints are slightly turned inward, whereas the pes imprints are oriented parallel to the midline or slightly rotated outward from the midline. The trackway shows an alternating arrangement of successive manus and pes imprints. Overprinting does not occur.

**Remarks:**

The small-sized tetradactyl manus imprints with short, thick digits ending in rounded terminations, and pentadactyl pes imprints are clearly assignable to the ichnospecies *Batrachichnus salamandroides*.

The *Batrachichnus* has been reliably documented from the Mississippian to the Middle Triassic of Europe (Makowsky & Rzehak, 1884; Fritsch, 1901; Pabst, 1908a; Pabst, 1908b; Haubold, 1970; Haubold, 1971; Haubold & Katzung, 1975; Gand, 1987; Voigt, 2005; Mujal et al., 2016; Marchetti, Avanzini & Conti, 2013; Marchetti et al., 2015b; Marchetti et al., 2020a; Marchetti et al., 2020b; Marchetti et al., 2022) North America (Lucas et al., 2011; Stimson, Lucas & Melanson, 2012; Voigt & Lucas, 2015a; Voigt & Lucas, 2015b; Voigt & Lucas, 2017; Klein & Lucas, 2021; Allen et al., 2022), South America (Melchor & Swithin Sarjeant, 2004), and Africa (Voigt et al., 2011a; Voigt et al., 2011b; Cisneros et al., 2020; Zouicha et al., in press).

**Table:** 

***Limnopus*** ** Marsh, 1894**
***Limnopus heterodactylus*** **(King 1845) (Figs. 8A–8D)**

**Referred material**. NPM P3573—convex hyporelief, manus-pes couple; CGS XA741—convex hyporelief, manus-pes couple

**Description:**

The manus imprints are tetradactyl and plantigrade, whereas the pes imprints are pentadactyl and plantigrade to semiplantigrade. The footprints are up to ∼30 mm in length. The manus imprints are about 1/3 wider than they are long, whereas the pes imprints are almost as long as wide. The manus imprint of the specimen NPM P3573 is roughly about half the size of the pes imprint (Table S1). The manus imprints are inwardly rotated compared with the pes imprint. The sole and palm impressions are rectangular in shape. Manual digit imprints are relatively short and thick. The manual and pedal digits show rounded terminations. The length of the manual digit imprints is similar and slightly increases from I to III, whereas digit IV is about as long as digit II. In the specimen NPM P3573, digit IV is separated from digits I–III and rotated outwards. The length of the pedal digits increases from I to IV, and V is shorter than III. The pedal digit impressions are in close vicinity to the manus imprint. The trackway shows an alternating arrangement of successive manus and pes imprints. The manus imprints show the most deeply impressed medial area within the tracks. Specimen ČGS XA741 (Figs. 8C–8D) shows distinct pads of digit I.

**Remarks:**

The specimens NPM P3573 and CGS XA741 have manus imprints noticeably wider than the total length of the footprint. The length of manual digits IV is as long as digit II in contrast with a very similar ichnotaxon *Batrachichnus*, which commonly has manus imprints as long as wide or slightly wider than they are long, and the manual digit IV is often shorter than digit II (Voigt, 2005). In addition, *Batrachichnus* pes imprints typically reach up to 20 mm in length. However, the distinct differences of digit proportions are not always clearly present. For this reason, some authors presented the *Batrachichnus*-*Limnopus* plexus (see Voigt et al., 2011a; Voigt et al., 2011b).

The specimen CGS XA741 (Figs. 8C–8D) was labelled as *Amphisauropus latus* = *A. kablikae* with the initial of H. Haubold, and this determination was used in the study by Haubold & Katzung (1975). We argue that the distinct pad impression of digit I was apparently mistaken for the digit I impression itself. The manual digits V of the *Amphisauropus* tracks are only slightly longer than the digits I (see Voigt, 2005; Voigt, 2015; Calábková, Březina & Madzia, 2022), which is inconsistent with the specimen CGS XA741. These facts most likely explain why the *Amphisauropus* tracks from the Ploužnice Horizon have never been described in subsequent studies. The morphology of the CGS XA741 is closely similar to that of *Limnopus* from the study of Voigt (2005).

*Limnopus* is well known from the Pennsylvanian to the Cisuralian of Europe (Tucker & Smith, 2004; Voigt, 2005; Voigt, 2015; Marchetti, Avanzini & Conti, 2013; Marchetti et al., 2015a; Marchetti et al., 2015b; Marchetti et al., 2020a; Niedźwiedzki, 2015; Meade, Jones & Butler, 2016; Mujal et al., 2016), Greenland (Milàn et al., 2016), North America (Martino, 1991; Van Allen, Calder & Hunt, 2005; Lucas & Dalman, 2013; Voigt & Haubold, 2015; Voigt & Lucas, 2015a), and North Africa (Voigt et al., 2011a; Lagnaoui et al., 2018).

**Table:** 

***Limnopus*** **isp. (Figs. 8E–8F)**

**Referred material.** MZM Ge34174—convex hyporelief, incomplete manus-pes couple.

**Description**

The large tetradactyl plantigrade manus imprint is ∼72 mm long. The manus and palm imprints are about 1/3 wider than they are long (Table S1). The digits are straight, thick, and short with rounded terminations. The digit IV impression is turned outward. The manual digits show almost the same length but increase slightly from I to III, while digit IV is almost the same length as digit II. The pes imprint is not completely preserved. The pedal digit imprints are thick, and relatively long with rounded terminations.

**Remarks**

**Figure 11:**
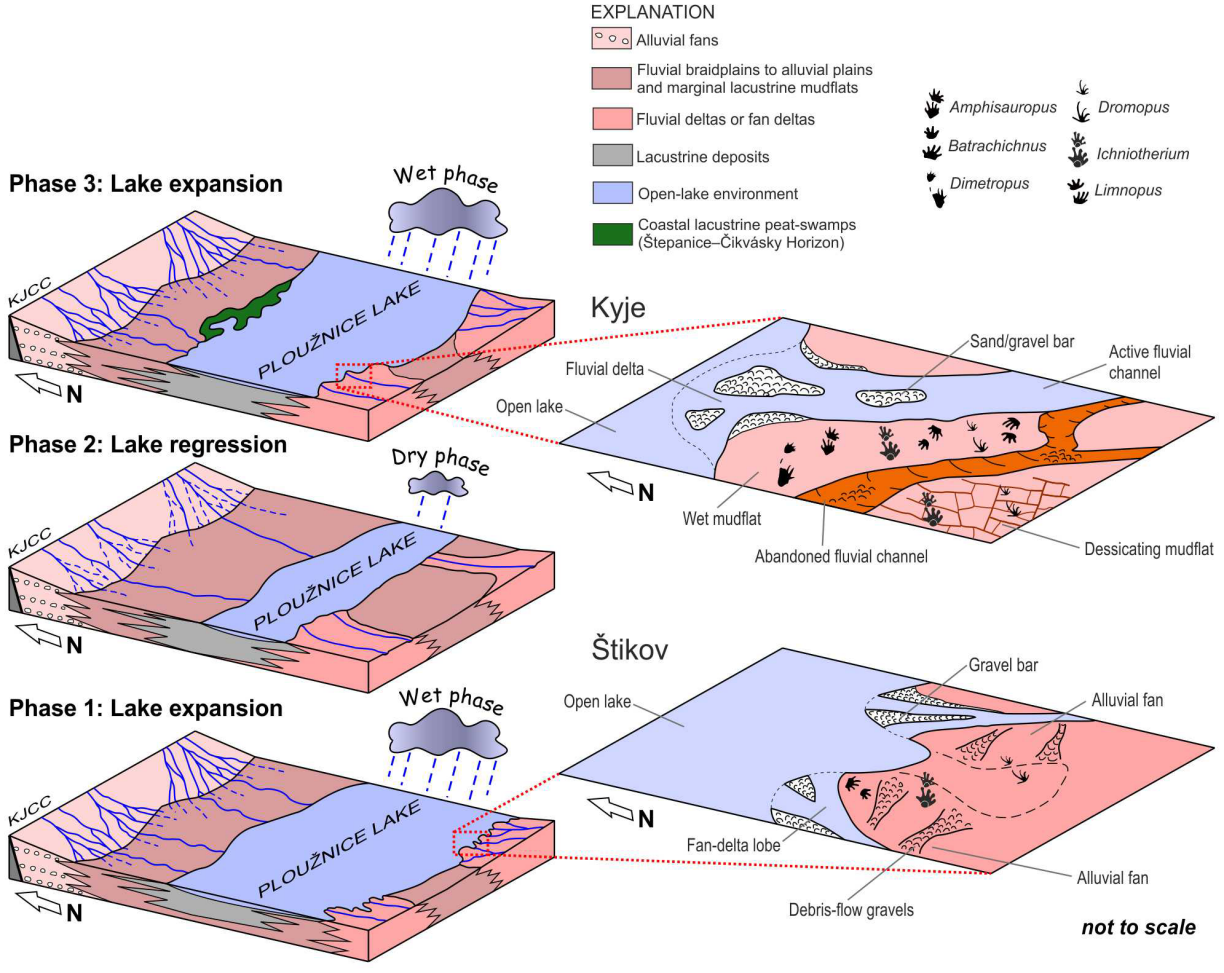
Blockdiagrams. A series of blockdiagrams illustrating the development (from the phase 1 to phase 3) of the Ploužnice Lake as interpreted from the studied sections (Kyje–Ploužnice railway cut, Štikov roadcut) and data on the Ploužnice Horizon by Blecha et al. (1997). The alternation of wet and dry climate phases that controlled the extent of the Ploužnice Lake, has already been interpreted from contemporaneous deposits of the Líně Formation in central Bohemia (Nádaskay et al., 2025). Close-ups provide a detailed interpretation of the depositional environment in which the trackmakers roamed and left their trace fossils.

The large tetradactyl manus imprints characterized by short, thick digits with rounded distal terminations, and rectangular palm impression correspond well to the large *Limnopus* tracks (Lucas & Dalman, 2013; Meade, Jones & Butler, 2016; Milàn et al., 2016; fig. 11). Regarding the incomplete pes impression, we assign the specimen MZM Ge34174 to *Limnopus* isp.

**Table:** 

***Ichniotherium*** ** Pohlig, 1892**
***Ichniotherium cottae*** **(Pohlig, 1885) (Figs. 9G–9H)**

**Referred material**. MZM Ge34184—convex hyporelief, manus-pes couple with two isolated tracks; CGS XA737—convex hyporelief, manus-pes couple; CGS XA739—hyporelief, isolated pes imprint; NPM P103—concave epirelief, isolated pes imprint; NPM P307—concave epirelief, isolated pes imprint.

**Description**

The manus and pes imprints are plantigrade and pentadactyl. The footprints are up to ∼135 mm long. The pes imprints are about 1/3 larger than the manus imprints. The pes imprints are slightly longer than wide or as wide as long, whereas the manus imprints are as wide as long, or wider than they are long (Table S1). The palm and sole impressions are deeply impressed with subcircular to elliptical shapes. The pedal digit imprints are rather straight and the manual digits II–IV are slightly bent inward. The digit terminations are distally extended and rounded, and they represent one of the most deeply impressed parts of the tracks. In the manus and pes imprints, the lengths of the digits increase from I to IV. The manual digits V are slightly shorter than the digits II or are of the same length. The pedal digits V reach approximately half the length of digit IV. The trackway shows an alternating arrangement of successive manus and pes imprints. No overprinting occurs within the manus-pes couple. The *Ichniotherium* track of NPM P307 (Figs. 9G–9H) is accompanied by *Dimetropus* manus-pes couple (Fig. 9I; see below for description) and small *Batrachichnus* tracks with tetradactyl manus impressions.

**Remarks**

The specimen MZM Ge34184, NPM P103, NPM P307, CGS XA737 and CGS XA 739 all show diagnostic features of *Ichniotherium cottae*, such as the relatively short pedal digit V with pV/pIV ratio < 0.60 (average value), deeply impressed elliptical to circular palm and sole, and deeply impressed distal parts of the digits (Voigt, Berman & Henrici, 2007). MZM Ge34184 (Figs. 9A–9B) shows a doubled sole impression, which was described in sole impressions of early Permian *I. cottae* from the Tambach Fm., Thuringian Forest, (see *e.g.*,Voigt, Berman & Henrici, 2007, fig. 3) as well as in palm impression of *I. cottae* from the Boskovice Basin, Czechia, in lowermost Permian deposits (Calábková et al., 2023a, fig. 2). *I. cottae* differs from *I. sphaerodactylum* in having a significantly shorter pedal digit V, in contrast to the longer digit V in *Ichniotherium sphaerodactylum*, which can reach about 80% of the length of pedal digit IV (see *e.g.*, Voigt, Berman & Henrici, 2007; Calábková et al., 2023a; Calábková, Ekrt & Voigt, 2023b). The CGS XA737 specimen (Figs. 9E–9F) preserves impressions of flexion creases on the digits, which are often present on *Ichniotherium cottae* tracks (*e.g.*, Voigt, 2005; Marchetti, Voigt & Santi, 2018; Calábková et al., 2023a).

The first occurrence of *I. cottae* comes from the Alveley locality, Birmingham, UK (Moscovian–Kasimovian) (Haubold & Sarjeant, 1973; Buchwitz & Voigt, 2018). Another Pennsylvanian occurrences come from the Gzhelian of Saar-Nahe Basin in Germany (Voigt, Berman & Henrici, 2007) and Ohio, USA (Baird, 1952). However, most of the *I. cottae* come from the Cisuralian of Europe (von Hochstetter, 1868; Frič, 1887; Fritsch, 1901; Haubold & Sarjeant, 1973; Haubold & Katzung, 1975; Voigt & Haubold, 2000; Voigt & Haubold, 2015; Voigt, 2005; Voigt & Ganzelewski, 2010; Voigt et al., 2012; Voigt et al., 2024; Mujal & Marchetti, 2020; Calábková et al., 2023a), USA (Voigt, Small & Sanders, 2005; Voigt & Lucas, 2015a; Voigt & Lucas, 2017), and North Africa (Lagnaoui et al., 2018).

**Table:** 

***Dimetropus*** ** Romer & Price, 1940**
***Dimetropus*** **isp. (Fig. 9I)**

**Material**. NPM P307—concave epirelief, manus-pes couple.

**Description**

The pes imprint is plantigrade, about 1/3 longer than it is wide, proximo-distally elongated, and ∼95 mm long (Table S1). The manus is imprinted only on its lateral part. The manus imprint seems distinctly smaller than the pes imprint. The manual and pedal digit impressions are relatively short, straight with a sharp clawed termination. The pedal digit impressions show a continuous increase in the length from I to IV, whereas the impressions of digits IV–V are not clearly identified. The impression of pedal digit I is orientated inwards. The manus-pes distance is relatively high.

**Remarks**

The elongated proximal part of the footprints, and relatively short and sharp terminated digit impressions are typical features for the ichnotaxon *Dimetropus leisnerianus* (Voigt, 2005). However, given the poorly preserved tracks and incompletely impressed manus, we assigned the track to *Dimetropus* isp., which shows great similarities with *Dimetropus* specimens from Morocco (Voigt et al., 2011b, fig. 5; Lagnaoui et al., 2014, fig. 5; Lagnaoui et al., 2018, fig. 6–7) and France (Gand, 1987, fig. 58A, planche 6B).

*Dimetropus* is known from the Pennsylvanian and the Cisuralian of Europe (Haubold, 1971; Gand & Haubold, 1984; Gand, 1987; Voigt, 2005; Niedźwiedzki & Bojanowski, 2012; Voigt & Ganzelewski, 2010; Voigt et al., 2012; Voigt & Lucas, 2015a; Voigt & Lucas, 2015b; Marchetti, 2016; Meade, Jones & Butler, 2016; Mujal et al., 2016; Matamales-Andreu et al., 2021; Matamales-Andreu et al., 2022; Calábková et al., 2023c; Marchetti et al., 2025), North America (Tilton, 1931; Haubold et al., 1995; Van Allen, Calder & Hunt, 2005; Sacchi et al., 2014; Voigt & Lucas, 2015a; Voigt & Lucas, 2015b; Lucas et al., 2016; Marchetti et al., 2020a), and North Africa (Voigt et al., 2011a; Voigt et al., 2011b; Lagnaoui et al., 2018). Except for the most common *Dimetropus leisnerianus,* the ichnospecies *Dimetropus osageorum* has been described from the Kungurian (lower Permian) of Oklahoma, USA (Sacchi et al., 2014), which differs from *D. leisnerianus* in that it shows a high degree of heteropody, short, subcircular manus imprints separated into two portions, short digit impressions which are subequal in length, and the pes imprint with a subelliptical to subcircular pad impression in the proximal central part of the sole. The morphological features of *D. osageorum* do not correspond to those of the specimen NPM P307.

**Table:** 

***Dromopus*** ** Marsh, 1894**
***Dromopus lacertoides*** **(Geinitz, 1861) (Figs. 10A–10F)**

**Referred material**. NM M4949a—concave epirelief, manus-pes couple and three isolated tracks; CGS XA735—convex hyporelief, three manus-pes couples and two isolated tracks; CGS XA742—convex hyporelief, isolated two tracks; CGS XA743—convex hyporelief, three manus-pes couples; CGS XA744—convex hyporelief, at least 8 tracks; NPM P104—convex hyporelief, manus-pes couple; NPM P3608—convex hyporelief, pes imprint; MZM Ge34176—convex hyporelief, manus-pes couples and two isolated tracks; MZM Ge34177—convex hyporelief and concave epirelief, at least 15 tracks; MZM Ge34178—convex hyporelief and concave epirelief, manus-pes couple; MZM Ge34179—concave epirelief, isolated track; MZM Ge34180—convex hyporelief and concave epirelief, manus-pes couple; MZM Ge 34181—convex hyporelief, manus-pes couple.

**Description**

The pentadactyl plantigrade to digitigrade manus and pes imprints of similar size and shape. The pes footprints are up to ∼70 mm long. The manus imprints are about 1/5 shorter than the pes imprints. The manus and pes imprints are longer than they are wide (Table S1). The manus imprints often show a slightly inward orientation to the midline, whereas the pes imprints show a parallel or slightly outward rotation to the midline. The impressions of the digits are long and slender with tapered terminations. Palm and sole impressions are short and often not impressed. The length of the digits increases from I to IV, and the digit V is about as long as digits II or III. Overprinting of the manus imprints by the pes imprints is common. The specimen NM M4949a (Figs. 10A–10B) shows also a several circular structures with a diameter between 3–5 cm.

**Remarks**

The manus and pes imprints, which are similar in shape and size, with long and slender digit imprints, correspond well to the ichnospecies *Dromopus lacertoides* (Voigt, 2005). The CGS XA742 footprints are among the largest *Dromopus lacertoides* which have been described to date. The *Dromopus* tracks are known from Late Pennsylvanian to late Permian deposits of Europe (Makowsky & Rzehak, 1884; Fritsch, 1901; Pabst, 1908a; Pabst, 1908b; Gand, 1987; Voigt, 2005; Voigt et al., 2012; Gand & Durand, 2006; Avanzini, Bernardi & Nicosia, 2011; Marchetti, Avanzini & Conti, 2013; Marchetti et al., 2015a; Marchetti et al., 2015b; Voigt & Haubold, 2015; Mujal et al., 2016), North America (Van Allen, Calder & Hunt, 2005; Lucas et al., 2011; Voigt & Lucas, 2015a; Voigt & Lucas, 2015b; Voigt & Lucas, 2017; Marchetti et al., 2020a), and North Africa (Voigt et al., 2011a; Voigt et al., 2011b). The circular structure on NM M4949a (Figs. 10A–10B) likely formed as a result of gas escaping from the sediment after it was compressed by passing tetrapods.

## Discussion

### Spatiotemporal significance of vertebrate ichnoassemblage

Tetrapod ichnofauna typically occurs earlier and has a wider palaeogeographic occurrence than the body fossils of their presumed trackmakers (*e.g.*, Niedźwiedzki et al., 2010; Long et al., 2025; Romilio et al., 2025). Therefore, the ichnological record plays a crucial role for tracing the spatiotemporal distribution of specific tetrapod groups, especially where the body fossils of terrestrial fauna are absent (Fig. 2). The tetrapod ichnoassemblage from the Semily Fm. revealed early occurrences of *Amphisauropus* tracks (ČGS JZ626, 632; Fig. 6) from the Ploužnice Horizon, which represents both the second oldest occurrence of this ichnotaxon worldwide, and the only thoroughly described *Amphisauropus* specimen from the Late Pennsylvanian. Although *Amphisauropus* was mentioned by Haubold & Katzung (1975) as originating from the Ploužnice Horizon (material stored at the Czech Geological Survey), this specimen was neither described nor figured in their study and most probably corresponds to the *Limnopus* track (CGS XA741) in our present study. The trackmakers of *Amphisauropus* are considered to be members of the Seymouriamorpha (Fichter, 1979; Marchetti, Mujal & Bernardi, 2016; Mujal & Marchetti, 2020), a group known primarily from lower Permian deposits in North America, Europe, and Asia (Amalitzky, 1921; White, 1939; Tchudinov & Vjuschokov, 1956; Tatarinov, 1968; Ivakhnenko, 1981; Berman, Reisz & Eberth, 1987; Berman & Martens, 1993; Klembara, 1995; Klembara, 2005; Klembara, 2009a; Klembara, 2009b; Sullivan & Reisz, 1999; Klembara & Batík, 2000; Bulanov, 2003; Klembara et al., 2020; Calábková, Březina & Madzia, 2022). The genus *Discosauriscus* is generally the most abundant representative of seymouriamorphs due to the hundreds to thousands of their larval stages discovered in the Letovice Fm. (Asselian) of the Boskovice Basin (location in Fig. 1A), Czechia. *Discosauriscus* larvae have also been found in the Prosečné Fm. (Asselian) of the KPB (Klembara et al., 2020). One of the oldest known *seymouriamorphs* is the discosauriscid *Utegenia shpinari,* which comes from the Kugaly Fm. in Kazakhstan (Kuznetsov & Ivakhnenko, 1981). This formation has been suggested to be either Pennsylvanian or Cisuralian in age (Kuznetsov & Ivakhnenko, 1981). Other early finds belong to *Discosauriscus* cf. *pulcherrimus* from the upper part of the Ilmenau Fm., which contains the Carboniferous–Permian boundary (Lützner et al., 2021; Klembara et al., 2023). Therefore, the identification of *Amphisauropus* tracks in the Late Pennsylvanian deposits of the KPB carries significant implications for understanding the spatiotemporal distribution of Seymouriamorpha. While skeletal remains of this group are predominantly known from the lower Permian strata across North America, Europe, and around the Urals (Amalitzky, 1921; White, 1939; Tchudinov & Vjuschokov, 1956; Tatarinov, 1968; Ivakhnenko, 1981; Berman, Reisz & Eberth, 1987; Berman & Martens, 1993; Klembara, 1995; Klembara, 2005; Klembara, 2009a; Klembara, 2009b; Sullivan & Reisz, 1999; Klembara & Batík, 2000; Bulanov, 2003; Klembara et al., 2020; Calábková, Březina & Madzia, 2022), our ichnofossil record, together with the *Amphisauropus* from the Middle Pennsylvanian of the USA (Lucas & Stimson, 2025; Marchetti et al., 2021), fundamentally support their earlier presence in the Pennsylvanian.

*Ichniotherium cottae* (MZM Ge34184; Figs. 9A–9B) from the Štikov, which falls into the lower part of the Semily Fm. (Stephanian C; Fig. 2) represents a rare occurrence of this ichnospecies from the European part of Pangaea in the Pennsylvanian. The morphology of *I. cottae* tracks and trackways closely corresponds to the structure of the autopodia and body posture of representatives of Diadectomorpha (see Voigt, Berman & Henrici, 2007; Buchwitz & Voigt, 2018). Although *Ichniotherium* is widespread in Permian deposits of the KPB (Frič, 1887; Frič, 1912a; Frič, 1912b) as well as in the nearby Intra-Sudetic Basin (Voigt et al., 2012; Voigt et al., 2024) and the Boskovice Basin (Calábková et al., 2023a; Fig. 1A), these tracks still represent the only evidence for the presence of diadectomorphs in the Pennsylvanian of Czechia. As diadectomorphs belong to one of the oldest lineages of herbivorous tetrapods, having evolved the ability to consume and process plant matter with a high fibre content (*e.g.*, Beerbower, Olson & Hotton, 1992; Hotton, Olson & Beerbower, 1997; Sues, 2000), their occurrence in a lake ecosystem extremely rich in flora (Fig. 2) is expected.

The studied *Limnopus* (Fig. 8) specimen represents the first record of this ichnotaxon from the territory of Czechia and thus expands our understanding of the distribution of large Pennsylvanian temnospondyls in the basins of the Central European Variscan Belt. While *Limnopus* trackmakers are generally accepted as medium to large-sized temnospondyls, particularly members of the Eryopidae clade (Baird, 1965; Gand, 1987; Haubold, 2000; Voigt, 2005), skeletal fossil records of such large temnospondyls in the KPB are rare and restricted only to younger Asselian strata (Fig. 2). These include eryopid and archegosaurid temnospondyls from the Vrchlabí Fm. (Steen, 1938; Milner, 1981; Štamberg & Zajíc, 2008) and an unspecified eryopid from the Prosečné Fm. (Štamberg, 2014).

Likewise, smaller *Batrachichnus* tracks (Fig. 7) indicate the presence of small-sized temnospondyls (Haubold, 1996; Voigt, 2005; Stimson, Lucas & Melanson, 2012) or even lepospondyls (Stimson, Lucas & Melanson, 2012; Allen et al., 2022). To date, no body fossils of lepospondyls have been discovered in the KPB, whereas Frič (1912a) and Frič (1912b) provided limited skeletal evidence of temnospondyls in his description of scarce remains of unspecified branchiosaurids from the Kyje–Ploužnice railway cut.

*Dimetropus* tracks are typically attributed to various non-therapsid synapsid groups, such as mostly carnivorous sphenacodontians and ophiacodotids, or herbivorous edaphosaurids, and caseasaurs (Tilton, 1931; Romer & Price, 1940; Haubold, 1971; Haubold, 2000; Fichter, 1979; Voigt, 2005; Niedźwiedzki & Bojanowski, 2012; Voigt & Ganzelewski, 2010; Sacchi et al., 2014; Romano, Citton & Nicosia, 2016). Skeletal evidence for early-diverging synapsids in Czechia is extremely rare and includes only *Macromerion schwarzenbergii*, discovered by Fritsch (1875) in the Pennsylvanian deposits of the Kounov locality (Gzhelian, Stephanian B) within the Slaný Fm., Kladno–Rakovník Basin (Fig. 1A). This locality also yielded an isolated dorsal vertebra of edaphosaurid *Bohemiclavulus mirabilis* (Fritsch, 1895). Additionally, an element of the dorsal spine of the edaphosaurid *Ramodendron obvispinosum* was discovered in the Gzhelian strata (Stephanian C) of the nearby Boskovice Basin (Švestka, 1944). Studied ichnofossils of *Dimetropus* (Fig. 9I) demonstrate the earlier presence of synapsids in the Pennsylvanian of the KPB.

*Dromopus* is undoubtedly the most abundant vertebrate ichnofossil in the Gzhelian deposits of the KBP. These tracks are referred to bolosaurid parareptile, aeroscelid diapsid or non-varanodontine varanopid trackmakers (*e.g.*, Haubold, 1971; Haubold, 2000; Voigt, 2005; Marchetti et al., 2021). Notably, no skeletal remains of provable early sauropsids or varanopids have been reported directly from KPB (Fig. 2). Only the unrevised material of potential sauropsids, tentatively assigned to “?*Macromerion*”, was figured by Fritsch (1885) from the Kounov locality (Gzhelian, Stephanian B) in the Kladno–Rakovník Basin. Additionally, the unrevised *Sphenosaurus sternbergii*, figured by Fritsch (1885), comes from an unknown locality in Bohemia, which Štamberg & Zajíc (2008) described as an “unknown Lower Permian locality (the red sandstone probably comes from the Krkonoše Piedmont Basin or from the Intra-Sudetic Basin)”. The extremely rich discoveries of *Dromopus* footprints from the KPB (Fig. 10) contribute significantly to our understanding of the initial diversification of amniotes, especially in areas where the fossil record of bodies is very limited.

Outside of the KPB, the only other Carboniferous tetrapod traces documented in Czechia come from the Radnice Member (Westphalian C, Pennsylvanian) of the Pilsen Basin, including *Gracilichnium* (?) *chlupaci* and *Lunichnium gracile*, interpreted as temnospondyl swimming, walking, and resting traces (Turek, 1989) and tetrapod footprints not described in more detail (Opluštil, Zajíc & Svoboda, 2022) from the Žacléř Formation (upper part of the Lampertice Member, Westphalian A, Pennsylvanian). Taken together, the tetrapod ichnoassemblage of the KPB is exceptionally crucial for understanding the diversity and evolution of terrestrial tetrapods in this intra-Variscan part of equatorial Pangea.

### Palaeoecological implications

The Štikov roadcut section is interpreted as deposits of a river mouth, possibly a braided delta, where individual gravel- or sand-filled distributary channels incised into violet-grey mudstones—*i.e.,* lacustrine nearshore deposits. The lacustrine deposits are considered here to result from the expansion of the Ploužnice Lake during a more humid period in the latest Pennsylvanian (Fig. 11)—a trend recorded by the contemporary Líně Fm. in central and western Bohemia (Nádaskay et al., 2025). The colour changes into red in upsection, which indicates lake-shore retreat and formation of a nearshore mudflat, accessible to the diadectomorphs, as well as eventually sauropsid and temnospondyl trackmakers.

The Kyje section (part of Kyje–Ploužnice railway cut) can be interpreted in terms of cyclic alternation of fluvial–alluvial and lacustrine environment of the so-called Ploužnice Lake (Fig. 11). The lowest of three identified cycles (cf. Blecha et al., 1997), with predominant reddish-brownish sediments, represents a vertical transition from lacustrine nearshore with intense clastic supply, probably close to a river mouth (Fig. 11) as evidenced by numerous sandstone bodies interpreted as mouth bars or alluvial bars, and one possibly incised alluvial or delta-plain channel. These are overlain by deposits of lacustrine coastal mudflat topped by a pedogenic horizon (Fig. 4B) that could be interpreted as a vertic calcisol. This indicates emergence of the lake coast for a longer period under a predominantly dry climate with seasonal precipitation (Fig. 11; cf. Mack & James, 1994). The overlying cycle is interpreted as a transition from the nearshore to the more distal portion of the lake. The tetrapod footprints were found in fine-grained sandstones in the lowermost part of this cycle, and were accompanied by mud drapes (sometimes with wrinkled surface possibly left by microbial mats), mudcracks, occasional rain-drop impressions, and invertebrate burrows (Figs. 5, 11). These features point to a sandy lake nearshore that repeatedly emerged for relatively short periods of time (cf. Melchor & Swithin Sarjeant, 2004; Minter et al., 2007; Mujal et al., 2016), inferred from the absence of significant rooting or pedogenic features. Similar environments can be interpreted from the topmost part of the Kyje section above the more distal lacustrine deposits. The footprints were left by early sauropsids, non-therapsid synapsids, diadectomorphs, seymouriamorphs, and temnospondyls, which roamed these areas during periods of low water level (no more than a few cm).

Concentric structures (Figs. 10A–10B) associated with *Dromopus* tracks, some of which overlie the tracks, are the most likely eroded subaerial parts of sand volcanoes that formed after the footprints. Formation of sand volcanoes is most commonly driven by dewatering related to earthquakes (*e.g.*, Montenat et al., 2007; Van Loon & Maulik, 2011), and it is possible that in this case they were generated by liquefaction of unconsolidated sand underlying the surface on which the trackmaker roamed. Build-up of pressure within the sandbed that preceded the “explosion” of a sand volcano (Owen, 1996) could have been ensured by a sealing mud layer covering the sandbed. Although sand volcanoes are among the frequently occurring structures of inorganic origin at vertebrate footprint sites (*e.g.*, Thulborn, 1990), their direct relationship to footprints—*i.e.,* their formation due to the loading of a sandbed by a passing trackmaker, has not been widely discussed. In contrast, other soft-sediment deformation structures have been interpreted as being produced by trackmakers (*e.g.*, Plint et al., 2025). The formation of sand volcanoes has also been attributed to the accumulation of gases from microbial mats (*e.g.*, Smith, Marsicano & Wilson, 2009; Taj, Aref & Schreiber, 2014), which were likely present at the margins of the Ploužnice Lake, as indicated by wrinkle structures on mud drapes.

By comparison, fossil-rich “bonebeds” are found within decidedly distal deposits of an open lake, since they contain bone fragments of predominantly aquatic (nektonic) vertebrates such as sharks, fishes and acantodes (Fig. 2). Sparse body fossils found in grey or violet–grey mudstones also provide evidence for a relatively open-lake environment, probably shallow and well-oxygenated (Blecha et al., 1997).

The sedimentary record of both studied localities allows for the interpretation of the lacustrine environment (Ploužnice Lake) that occupies the central part of the basin, fed by fluvial systems entering the basin from the south, forming fan deltas (Štikov section) or more widely distributed fluvial deltas on a flatter basin margin (Kyje section). We assume that the evolution of the Ploužnice Lake (Fig. 11) was strongly influenced by relatively short-term (100–400 Kyr) climate fluctuations reflected by the decrease and increase in precipitation, as suggested by the evolution of a coeval depositional system of the Líně Fm. (with Klobuky Lake) in central Bohemia (Nádaskay et al., 2025).

The abundance of footprints in the Semily Fm. suggests that the lake ecosystem was a sought-after habitat for terrestrial and semi-terrestrial tetrapods, whether as a source of water, food, or a place for reproduction. The occurrence of *Limnopus* tracks, most probably left by large temnospondyls, corresponds well with the abundant fish fossils (Fig. 2), as representatives of eryopids and stereospondylomorphs were mostly piscivorous (Boy, 2003; Schoch, 2021). The diverse plant assemblage (Fig. 2) provided a rich food source for all herbivores such as diadectomorphs, indicated by *Ichniotherium* tracks. It remains unclear whether the *Dimetropus* tracks were left by herbivorous or carnivorous synapsids. In any case, the studied Pennsylvanian ecosystem of the KPB provided sufficient prey for apex predators of the Late Carboniferous such as synapsids. Finally, the abundance of fossil insects (Fig. 2) demonstrates good food availability for early sauropsids, as well as for many varanopids, which are represented by their relatively rich *Dromopus* track record.

The most complete data on fossil biota are provided by the lacustrine fossiliferous horizons within the KPB, where conditions for fossil preservation were most favourable. The Pennsylvanian taxonomic diversity likely persisted across the Carboniferous-Permian boundary, as the taxonomic richness of the fossil assemblages in the Semily Fm. appears comparable to that of the Vrchlabí Fm. (Fig. 2). During the early Permian, after deposition of the Rudník Horizon (Vrchlabí Fm., lowermost Asselian), a slight decline in the taxonomic richness of the fossil record is observable within the following Kalná Horizon (Prosečné Fm., Asselian). However, the question remains whether this apparent decline reflects a real reduction in palaeodiversity or is the result of taphonomic bias. Only a comprehensive analysis of the fossil record from Permian formations in the KPB can provide adequate data.

## Conclusions

The uppermost Carboniferous deposits of the Semily Fm. (Gzhelian, Stephanian C) in the KBP have revealed the presence of six ichnotaxa, assigned to five ichnospecies *Amphisauropus kablikae*, *Batrachichnus salamandroides*, *Dromopus lacertoides*, *Ichniotherium cottae*, and *Limnopus heterodactylus*, and two unknown ichnospecies *Dimetropus* isp. and *Limnopus* isp. These ichnotaxa are attributed to seymourimorphs, temnospondyls, diadectomorphs, early sauropsids and non-therapsid synapsids, which fill critical gaps given by the extremely sparse body fossil record, providing invaluable insights into the faunal composition and ecological dynamics of early tetrapod ecosystems during this pivotal period in evolution of terrestrial tetrapods.

Late Paleozoic limnic basins, such as the KPB, were likely crucial for the survival of terrestrial and semi-terrestrial tetrapods within the continental interior environment of the Variscan Belt. Fluctuations in the lake water level played a key role in preserving their footprints, which often represent the only direct evidence of their presence in this area. The diversity of ichnotaxa makes this site the richest locality for Upper Pennsylvanian tetrapod footprints. Palaeoecosystems in the KPB appear to have persisted without major changes until the beginning of the early Permian.

##  Supplemental Information

10.7717/peerj.20437/supp-1 Supplemental Information 1 Track parameters Measurements (in mm) obtained from the selected studied fossil tracks. Abbreviations: L = length of track, W= width of track, I–IV = length of digits I–V, *track parameters were calculated as average values. All specimens were measured using a manual calliper. Specimens MZM Ge34174, MZM Ge34176, and MZM Ge34184 were remeasured from photographs using ImageJ software.

## Abbreviations

CGS: Czech Geological Survey, Prague, Czechia
MZM: Moravian Museum, Brno, Czechia
NM: National Museum, Prague, Czechia
NPM: Nova Paka City Museum, Nová Paka, Czechia

## Additional Information and Declarations

### Data Availability

The following information was supplied regarding data availability: The raw data is available in the Supplemental File.
